# Zn^2+^-dependent association of cysteine-rich protein with virion orchestrates morphogenesis of rod-shaped viruses

**DOI:** 10.1371/journal.ppat.1012311

**Published:** 2024-06-17

**Authors:** Ning Yue, Zhihao Jiang, Qinglin Pi, Meng Yang, Zongyu Gao, Xueting Wang, He Zhang, Fengtong Wu, Xuejiao Jin, Menglin Li, Ying Wang, Yongliang Zhang, Dawei Li

**Affiliations:** 1 State Key Laboratory of Plant Environmental Resilience, College of Biological Sciences, China Agricultural University, Beijing, China; 2 College of Plant Protection, China Agricultural University, Beijing, China; University of Cambridge, UNITED STATES

## Abstract

The majority of rod-shaped and some filamentous plant viruses encode a cysteine-rich protein (CRP) that functions in viral virulence; however, the roles of these CRPs in viral infection remain largely unknown. Here, we used barley stripe mosaic virus (BSMV) as a model to investigate the essential role of its CRP in virus morphogenesis. The CRP protein γb directly interacts with BSMV coat protein (CP), the mutations either on the His-85 site in γb predicted to generate a potential CCCH motif or on the His-13 site in CP exposed to the surface of the virions abolish the zinc-binding activity and their interaction. Immunogold-labeling assays show that γb binds to the surface of rod-shaped BSMV virions in a Zn^2+^-dependent manner, which enhances the RNA binding activity of CP and facilitates virion assembly and stability, suggesting that the Zn^2+^-dependent physical association of γb with the virion is crucial for BSMV morphogenesis. Intriguingly, the tightly binding of diverse CRPs to their rod-shaped virions is a general feature employed by the members in the families *Virgaviridae* (excluding the genus *Tobamovirus*) and *Benyviridae*. Together, these results reveal a hitherto unknown role of CRPs in the assembly and stability of virus particles, and expand our understanding of the molecular mechanism underlying virus morphogenesis.

## Author summary

The symmetrical structures of plant RNA viruses are usually composed of numerous identical coat protein (CP) subunits encapsidated one molecule of viral genomic RNA. Interestingly, a large number of rod-shaped and filamentous plant viruses encode a cysteine-rich protein (CRP), which usually function as viral suppressors of RNA silencing and pathogenicity determinants during viral infection. However, their roles in viral life cycle remain poorly understood. Here, our study reveals the essential role of CRPs in virus morphogenesis. The cysteine-rich γb protein binds to the surface of rod-shaped barley stripe mosaic virus (BSMV) virion by interacting with CP in a Zn^2+^-dependent manner, thereby facilitating virion assembly and stability. Both His-85 site in γb predicted to generate a potential CCCH motif and His-13 site in CP exposed to the surface of the virions are key amino acids in γb-CP interaction and the zinc-binding activity. Attractively, the phenomenon of CRPs being tightly bound to their rod-shaped virions occurs in a broad range of distantly related viruses, implying a conserved role for the CRPs in orchestrating virus morphogenesis.

## Introduction

Viral particles usually present helical or spherical (icosahedral) symmetry. Plant viruses with helical symmetry can be further divided into rod-shaped viruses, represented by the families *Virgaviridae* and *Benyviridae* [1,2], while flexuous filamentous virions can be found in the families *Alphaflexiviridae*, *Betaflexiviridae*, *Gammaflexiviridae*, *Closteroviridae*, and *Potyviridae* [3–5]. The morphology differences of the helical virion subdivision between rod-shaped and filamentous viruses are mainly caused by the structural folds of coat proteins (CPs) and inter-subunit interactions of CP monomers [3–7]. Apart from facilitating virion assembly and ensuring the stability of viral genomes [3], CP proteins serve diverse functions in viral infectivity, pathogenicity, vector transmission, host range determination as well as counteracting antiviral responses [8,9]. Interestingly, a large number of rod-shaped and filamentous plant viruses encode a cysteine-rich protein (CRP) [10–14]; however, their functions in viral infection, especially in virion morphogenesis, remain to be elucidated.

Besides the structural protein CP and nucleic acids, some viral nonstructural proteins, host factors, and small molecules are also required to virion assembly and disassembly [7,8]. Plum pox virus (PPV) HC-Pro and P3 proteins are essential for the formation of stable virions [15,16], potato virus X (PVX) TGB1 binds to one end of its helical particle and triggers the disassembly into a translatable form [17,18]. Both the CP readthrough proteins of potato mop-top virus (PMTV) and beet necrotic yellow vein virus (BNYVV) associate with one extremity of the virus particles, to improve PMTV long-distance movement [19,20] or BNYVV virions assembly [21]. Moreover, the cell-to-cell movement of bamboo mosaic virus (BaMV) requires the stable association of virion with the TGB2- and TGB3-based membrane complex [22]. As for the host factor, heat shock cognate 70-kDa protein (Hsc70) is physically associated with cucumber necrosis virus (CNV) icosahedral virions to reduce the stability of CNV virions, thereby releasing the encapsidated viral genome to initiate new infection [23].

Metal ions, represented by zinc, also play vital roles in virus packaging, inhibiting the replication of RNA viruses, antiviral immunity, and host-virus interactions [24–26]. The structural protein Gag precursor of human immunodeficiency virus type 1 (HIV-1) participates in the assembly of budding virion, the nucleocapsid (NC) domain of Gag binds to the 3’-leader of the genomic RNA to initiate the packaging of HIV virions [27]. The NC domain or its cleaved version contains two CCHC zinc-finger motifs, mutations of these zinc-finger motifs cause severe defects in viral RNA packaging and infectivity [27,28]. Zn^2+^ mediates the conformational changes of hepatitis B virus (HBV) CP to increase the production of virus-like particles (VLPs) [29]. In plants, the zinc finger domain in tobacco streak virus (TSV) CP is required for the assembly of VLPs, and the stability of TSV particles is significantly increased in the presence of Zn^2+^ [30].

*Barley stripe mosaic virus* (BSMV) is the prototypical member of the genus *Hordeivirus* (family of *Virgaviridae*), the rod-shaped virions encapsidated tripartite plus-strand RNAs designated RNAα, RNAβ, and RNAγ [1,31,32]. RNAα and RNAγ encoding the viral replicase subunits αa and γa are sufficient to support viral replication. The CP, which is translated directly from the first RNAβ ORF, is associated with viral genomic RNAs (gRNAs) for virion encapsidation. The RNAβ also encodes triple gene block proteins (TGB1, TGB2, and TGB3) for viral cell-to-cell movement. The cryo-electron microscopy structure of BSMV particles at 4.1 Å found two different sizes of virions [33]. The wide form virions contain 111 CP subunits per helix period, while the helical parameters of narrow virions are 106, suggesting that the conformation of CP subunits and the interaction with genomic RNAs are different in the two forms. A series of structural data show that the virions of narrow form are more stable than the wide versions due to the stronger inter-subunit contact between different CP subunits or with viral RNAs [3,33]. However, mechanisms governing the regulation of wide and narrow particles remain to be elucidated, and whether other factors and/or small molecules exist on the virus particles and function in BSMV morphogenesis remains to be determined.

The 17 kDa cysteine-rich γb protein encoded by BSMV RNAγ has a versatile role in viral replication [34], movement [35], and interactions with distinct host factors [32,36–39]. In this study, we found that γb specifically binds to the purified rod-shaped BSMV particles in a Zn^2+^-dependent manner. The association of γb with CP is required for the stability and morphogenesis of BSMV virions. In addition, we also provide evidence that the physical association of the CRPs with the rod-shaped virions is a general feature among the members in the families *Virgaviridae* and *Benyviridae*. These results reveal a hitherto unknown role of CRPs during viral infection cycle and deepen our understanding of the molecular mechanism underlying virus morphogenesis.

## Results

### γb is associated with BSMV virions

To identify the host proteins associated with BSMV virions, virus particles were purified from systemically infected leaves of *Nicotiana benthamiana* using a similar strategy to one described previously [23]. Mock-inoculated plants served as negative controls. The purified BSMV virions and the products purified from mock-inoculated plants were separated by 12.5% SDS-PAGE gel, followed by silver staining. The visible gel bands that were present in the purified virions but absent in the negative control were cut and divided into four groups (S1 Fig), followed by Q-Exactive liquid chromatography–tandem mass spectrometry (LC-MS/MS) analysis. In line with our expectations, we identified a large number of host factors that could be associated with BSMV virions. Surprisingly, the MS results showed that γb proteins were detected in all four groups (S1 Table). Previously, we found that CPs co-precipitated with γb-3xFlag proteins in the IP products from BSMV_γb-3xFlag_-infected *N*. *benthamiana* leaves [35]. These results suggest that γb may be associated with CP *in vivo* in the context of BSMV infection.

**Fig 1:**
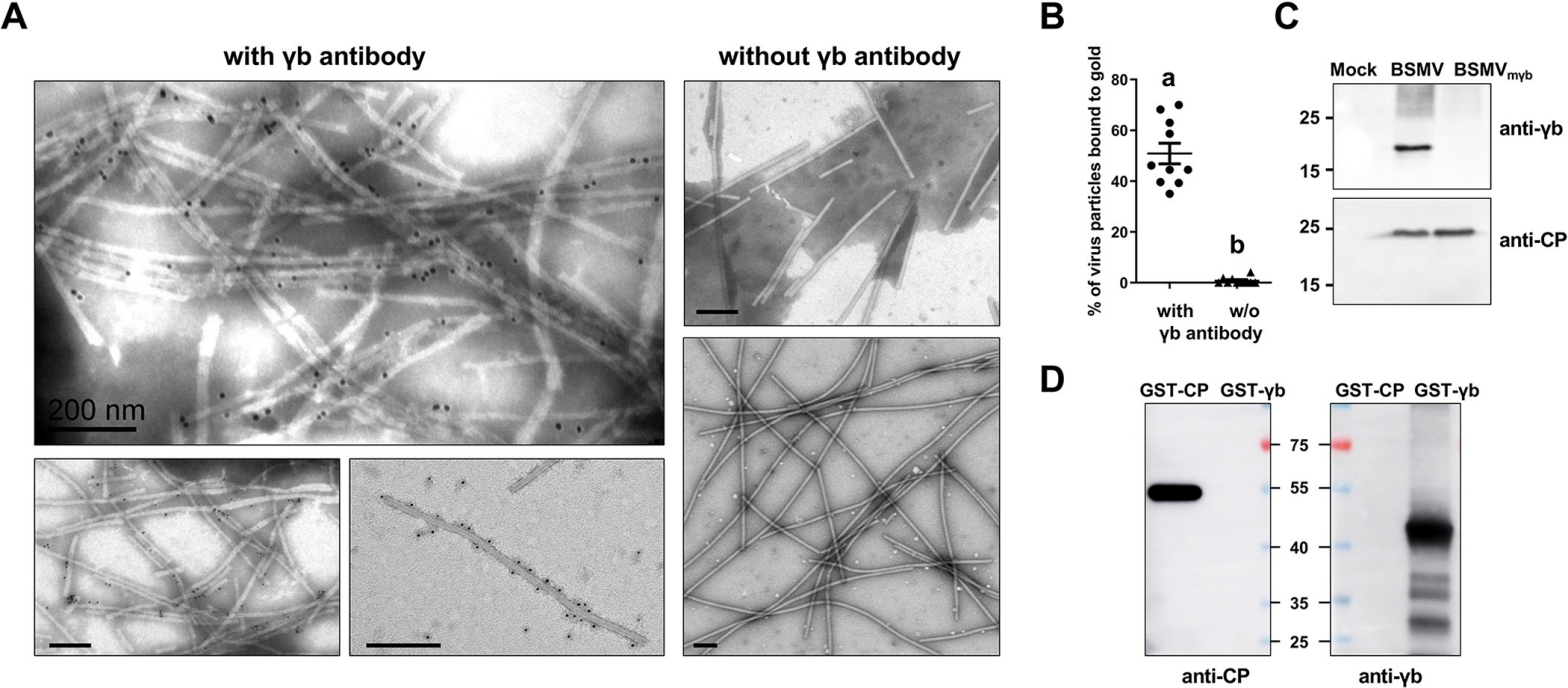
γb protein is associated with the surface of purified BSMV virions. **(A)** Representative immunogold labeling images showing γb protein bound to purified BSMV virions (left three photos), the labeling without γb antibody was used as a negative control (right two photos). Representative data are shown and the experiments have done more than three biological replicates with similar results. Scale bar, 200 nm. **(B)** The average amount of γb proteins associated with purified BSMV virions as shown in Fig 1A. Different letters above the bars indicate statistically significant differences (*p* < 0.05) as determined by Duncan’s multiple range test (*n* = 10). **(C)** Western blot to detect the target protein expression in BSMV-infected *N*. *benthamiana* leaves with CP and γb antiserum.The BSMV_mγb_ mutant has a UUG to AUG substitution to destroy the translation of γb. **(D)** Detection of the cross-reactivity between γb and CP antibodies. GST-CP and GST-γb were purified followed by Western blot using CP and γb antibodies.

To determine whether γb directly binds to BSMV virions, immunogold labeling experiments were performed (Fig 1A and 1B). Purified BSMV virions were adsorbed onto Formvar/carbon-coated nickel grids, followed by incubation with γb antibody or BL buffer. After labeling by goat anti-rabbit secondary antibody conjugated to 10 nm colloidal gold particles, the samples were observed by transmission electron microscopy (TEM). The results showed that approximately 50.87% of virions were specifically labeled with gold particles on the surface; in contrast, only a few gold particles were associated with the virions incubated with BL buffer or the pre-immune rabbit serum (Figs 1A and 1B and S2A), and we conducted immunogold labeling experiments with BSMV γb polyclonal antibody in the purified viral particles of lychnis ringspot virus (LRSV; genus *Hordeivirus*), poa semilatent virus (PSLV; genus *Hordeivirus*), tobacco rattle virus (TRV; genus *Tobravirus*), tobacco mosaic virus (TMV; genus *Tobamovirus*), and BSMV simultaneously. Notely, only the BSMV virions exhibited abundant binding with gold particles (S2B Fig). These results indicate that γb is associated with the outer surface of BSMV virions.

To clarify the specificity of γb antiserum, we tested the cross-reactivity of BSMV CP protein with the γb antiserum by using BSMV-infected *N*. *benthamiana* leaf samples or its mutant BSMV_mγb_ (an AUG→UUG substitution of the γb initiation codon), as well as GST-CP and GST-γb proteins [35], the results demonstrated that the γb antiserum is highly specific and does not cross-react with the CP protein (Fig 1C and 1D).

Altogether, these results indicate that γb interacts with and locates to the surface of BSMV virions.

### γb interacts with CP *in vivo* and *in vitro*

**Fig 2:**
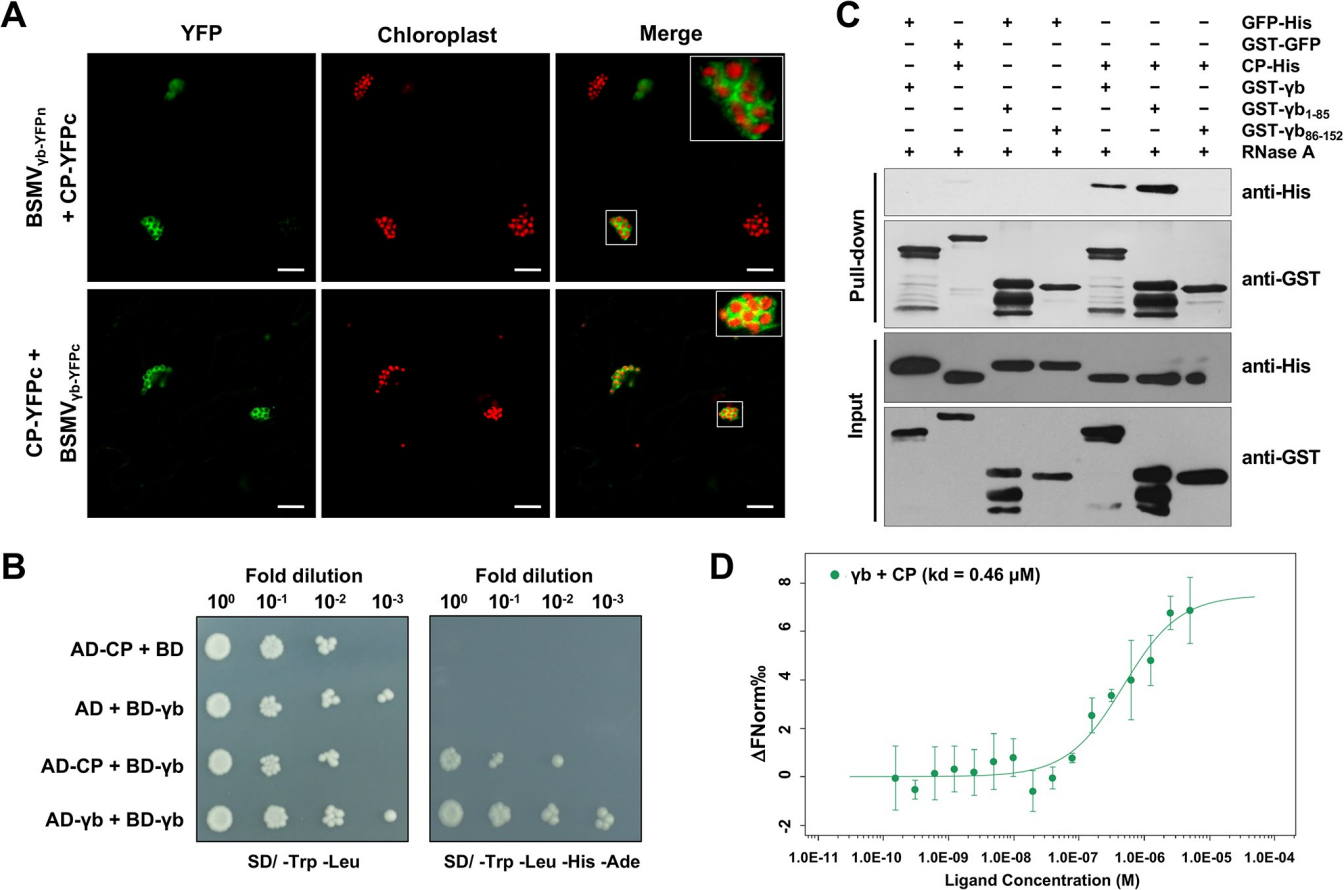
γb interacts with CP *in vivo* and *in vitro*. **(A)** Interaction of γb with CP in the context of BSMV infection by using BiFC assay. CP-YFPn or CP-YFPc was co-expressed with BSMV_γb-YFPn_ or BSMV_γb-YFPc_. Chloroplast autofluorescence is shown as false red color. The negative control of BiFC assay is shown in S3 Fig. Scale bars, 20 μm. **(B)** Yeast two-hybrid (Y2H) assay to analyze the interaction between CP and γb. γb was cloned as a translational fusion with BD, CP was cloned as a translational fusions with AD, and combinations containing empty AD or BD constructs were served as negative control. Serial 10-fold dilutions of liquid cultures were spotted on synthetic dextrose dropout medium, SD/-Trp-Leu or SD/-Trp-Leu-His-Ade. **(C)** GST pull-down assay for the interaction between CP and γb *in vitro*. Various γb mutants were fused with GST-tag and incubated with CP-His with or without the 10 μg RNaseA treatment. GST-GFP or GFP-His was used as a negative control. The immunoprecipitated proteins were analyzed by Western blot analysis using anti-GST or anti-His antibodies. **(D)** Validate the interaction between CP and γb by MST assay. The concentration of γb-GFP-His was held constantly at 10 μM and the concentration of CP-His was titrated from the 20 μM. MST measurements were performed at 25°C by using 20% LED-power and 40% MST-power. Data analyses were used the MO. Affinity Analysis (x86) software. The dissociation constant (K_d_) was derived to be K_d_ = 0.46 μM.

To test whether the association of γb with BSMV virions depends on the CP–γb interaction, we performed a BSMV-based biomolecular fluorescence complementation (BiFC) assay [35]. Recombinant yellow fluorescent protein (YFP) signals were observed at the periphery of chloroplasts in *N*. *benthamiana* epidermal cells co-expressing either CP-YFPn with BSMV_γb-YFPc_ or CP-YFPc with BSMV_γb-YFPn_ (Figs 2A and S3). The yeast expressing AD-CP and BD-γb grew well on SD/-Trp-Leu-His-Ade drop-out plates (Fig 2B), indicating that CP interacts with γb in Y2H system. The GST pull-down result showed that both GST-γb and GST-γb_1-85_ directly pulled down CP-His, indicating that the N-terminal of γb (aa 1–85) is sufficient for its interaction with CP *in vitro* (Fig 2C).

To further confirm the interaction between CP and γb further, we performed microscale thermophoresis (MST) measurements. The concentration of γb-GFP-His protein was kept constant at 10 μM, and different amounts of CP-His protein were titrated starting from 20 μM. The dissociation constant (K_d_) was derived as K_d_ = 0.46 μM (Fig 2D), suggesting a strong affinity between γb and CP proteins.

Taken together, these results showed that γb protein directly interacts with CP *in vivo* and *in vitro*, and the N-terminus of γb is key motif that interacting with CP protein.

### γb interacts with CP in a Zn^2+^-dependent manner

**Fig 3:**
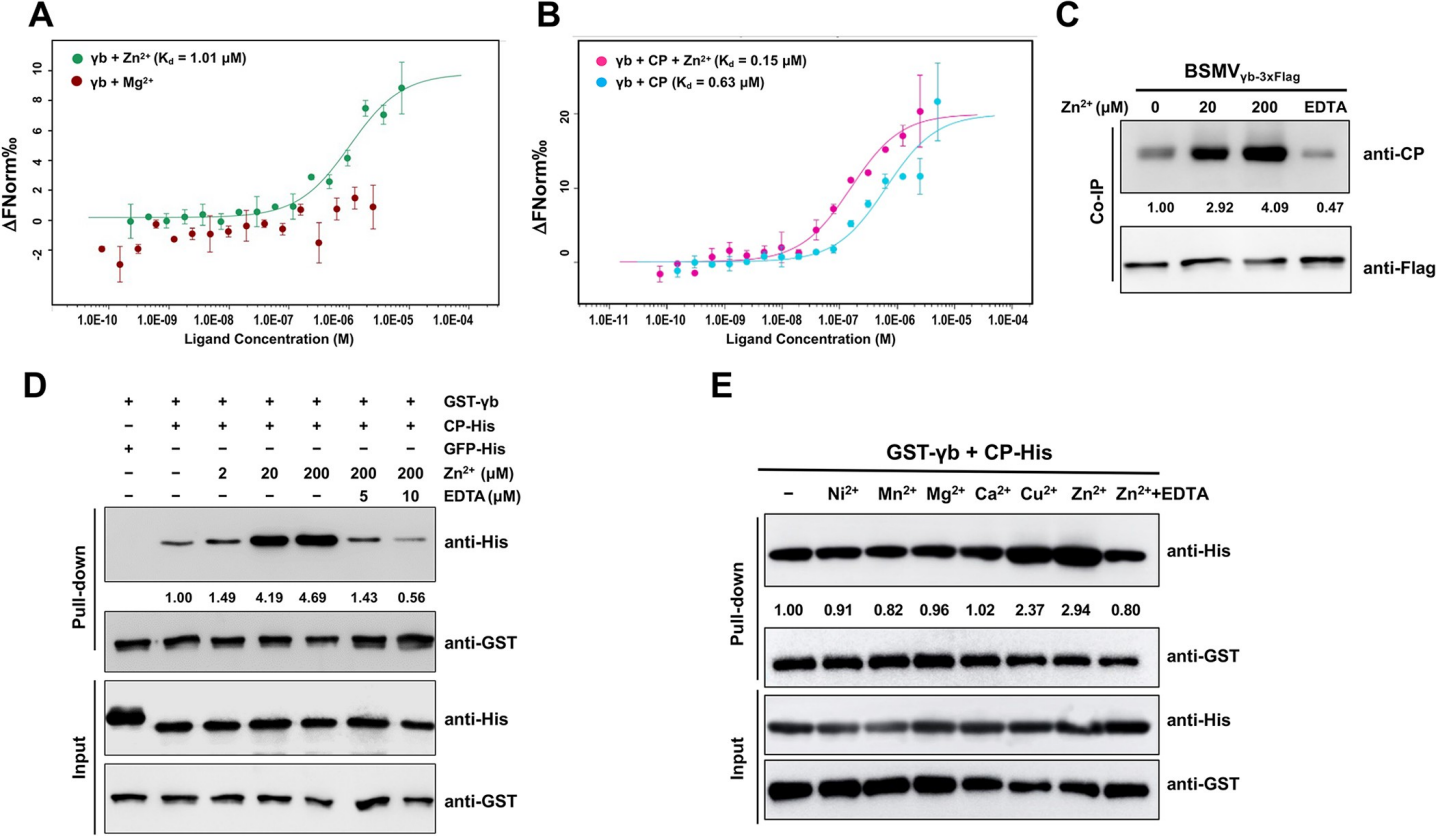
The interaction between γb and CP is specifically enhanced by Zn^2+^ *in vivo* and *in vitro*. **(A)** MST assays showed that γb binds to the Zn^2+^. The γb-GFP-His protein was purified from *E*. *coli*. In this assay, the concentration of γb-GFP-His was consistent, while 5 μM Zn^2+^ or Mg^2+^ were used as ligands. The apparent dissociation constant between γb and Zn^2+^obtained was K_d_ = 1.01 μM. The obtained data were digitized using a Monolith NT.115 instrument. **(B)** MST assays showed that the interaction between γb and CP was enhanced by Zn^2+^. The γb protein alone or the γb protein containing 5 μM Zn^2+^ in the buffer was kept constant and the CP protein was added as a ligand. In the absence of Zn^2+^, γb was bound to the CP membrane with K_d_ = 0.63 μM. In the presence of Zn^2+^, the affinity increased 4-fold with K_d_ = 0.15 μM. The resulting data were digitized with Monolith NT.115 instrument. Error bars indicate the range of data points obtained from at least two measurements. **(C)** Zn^2+^ enhances CP-γb interaction by Co-IP assay. *N*. *benthamiana* leaf tissues co-agroinfiltrated with RNAα, RNAβ, and RNAγ_γb-3xFlag_ were harvested at 3 dpi. Various concentrations of Zn^2+^ were added to the crude protein extracts. Total proteins were immunoprecipitated with anti-Flag beads. Input and immunoprecipitated protein (IP) were analyzed by Western blot analysis with an anti-GFP or anti-Flag antibody. **(D)** Zn^2+^ can enhance the interaction between γb and CP by GST Pull-down assay. Purified GST-γb and CP-His were incubated with a concentration gradient of Zn^2+^ from 2 μM to 200 μM. Various concentrations of EDTA were used to destroy the zinc-binding activity. Western blot analysis of immunoprecipitated proteins was performed using anti-GST or anti-His antibodies. **(E)** Zn^2+^ specifically enhances the interaction between γb and CP. The concentration of divalent metal cations (Cu^2+^, Ni^2+^, Mn^2+^, Mg^2+^, Ca^2+^, and Zn^2+^) was 20 μM.

Previous studies have shown that the N-terminal (aa 1–85) of γb contains three zinc-binding motifs [40], but the function of the zinc-binding motifs remains largely unknown. Since the N-terminal of γb interacts with CP (Fig 2C), we hypothesized that the zinc-binding activity may be functionally linked to the γb–CP interaction. The MST assay demonstrated the Zn^2+^-binding activity of γb, but it did not bind to Mg^2+^ (Fig 3A). Next, MST, co-IP, and GST pull-down assays were performed to identify the effect of Zn^2+^ on the γb-CP interaction, all these results showed that the binding affinity of γb and CP was enhanced by the addition of Zn^2+^ *in vitro* and *in vivo* (Fig 3B–3D). In contrast, the divalent cation chelator EDTA substantially impaired the CP–γb interaction in the presence of 200 μM Zn^2+^ (Fig 3C and 3D). Moreover, the addition of Zn^2+^ and Cu^2+^ increased the interaction between CP and γb, whereas other bivalent ions had no effect (Fig 3E). Together, these results suggest a positive regulatory role of Zn^2+^ in the CP–γb interaction.

### γb is physically associated with virions in a Zn^2+^-dependent manner

**Fig 4:**
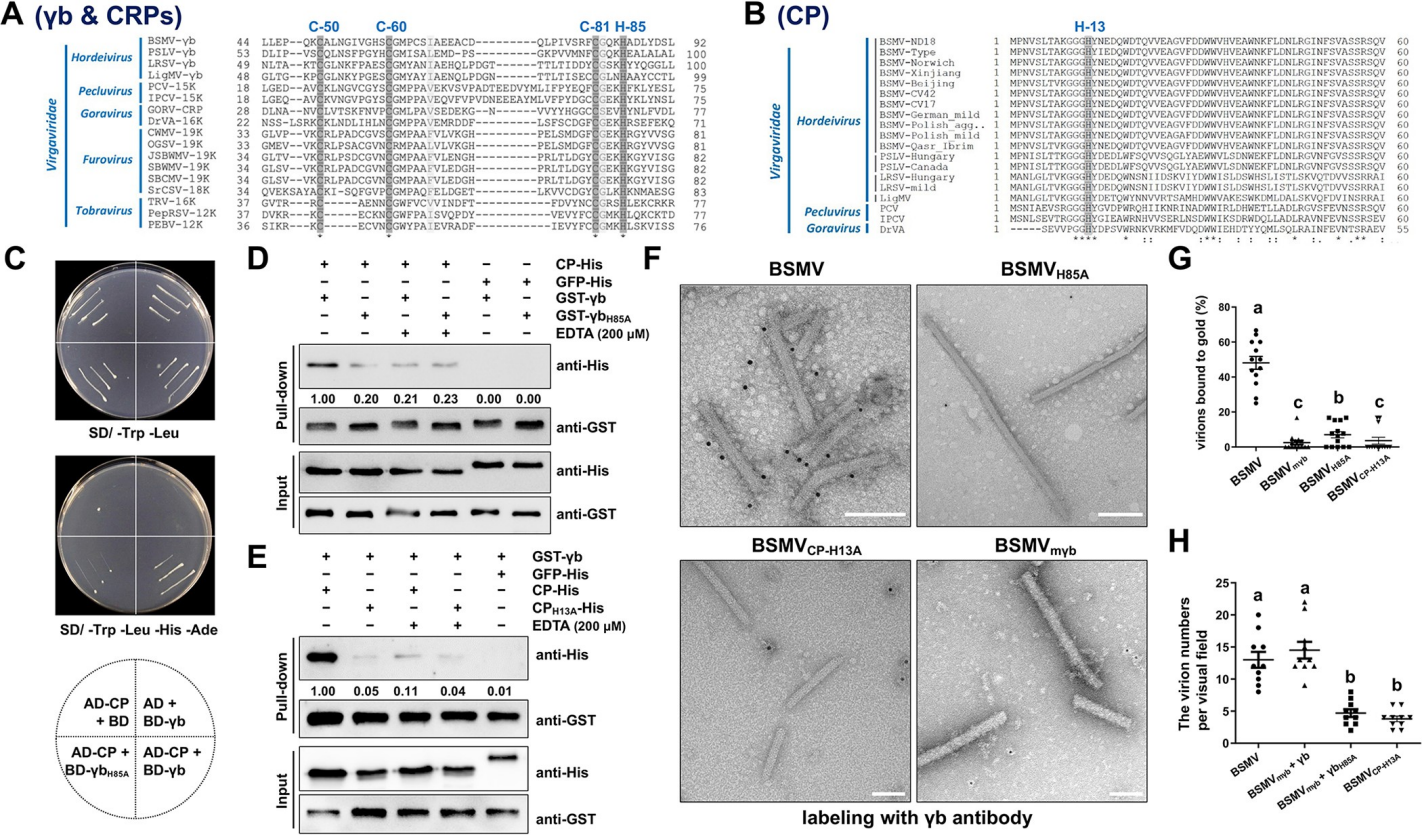
γb physically associates with virions by interacting with CP in a Zn^2+^-dependent manner. **(A)** Amino acid sequence alignment of CRP proteins in different genera of *Virgaviridae*. Sequence alignment was performed by using the Uniprot online server (https://www.uniprot.org/). The conserved CCCH motif (Cys-50, Cys-60, Cys-81 and His-85 for BSMV strain ND18) are highlighted in gray. **(B)** Amino acid sequence alignment of CPs in different genera of *Virgaviridae*. The N-terminal of CPs among *Virgaviridae* were analyzed by using the Uniprot online server. The conserved His 13 of BSMV strain ND18 is highlighted in gray. **(C)** Yeast two-hybrid (Y2H) assay to analyze the interaction between CP and γb_H85A_. γb_H85A_ was cloned as a translational fusion with BD. The interaction between γb and CP was served as a positive control. Combinations containing the empty vector pGADT7 or pGBKT7 were used as negative controls. Yeast cells containing the indicated plasmids in the bottom panel were spotted on synthetic dextrose dropout medium, SD/-Trp-Leu or SD/-Trp-Leu-His-Ade. **(D and E)** His-85 of γb and His-13 of CP are required for zinc binding activity and interaction between CP and γb. γb and γb_H85A_ were fused to GST-tag and incubated with CP-His or CP_H13A_-His. 200 μM EDTA was used to destroy zinc-binding activity in the GST pull-down assays. GFP-His was used as a negative control. Western blot analysis of immunoprecipitated proteins was performed using anti-GST or anti-His antibodies. **(F)** Immunogold labeling experiments showed that γb binds to BSMV virions by interacting with CP in a Zn^2+^-dependent manner. BSMV and its derivatives virions were purified from infected leaves of *N*. *benthamiana* at 5 dpi. Virions were adsorbed onto 200-mesh nickel grids and incubated with antibodies against the γb protein. The pictures were visualized by TEM at 80 kV. Scale bar, 100 nm. **(G)** Average amounts of γb protein that are associated with the surface of purified BSMV virions as shown in Fig 4F. Different letters above the bars denote statistically significant differences (*p* < 0.05) determined by the Duncan’s multiple range test (*n* = 13). **(H)** Quantification of the average number of virus particles. BSMV, BSMV_mCP_, and BSMV_mγb_ were agroinfiltrated into *N*. *benthamiana* leaves. γb-3xFlag or γb_H85A_-3xFlag was expressed with BSMV_mγb_ infectious cDNA clone. The virions of different derivatives were purified at 5 dpi. Different letters in the chart denote statistically significant differences among different groups according to the Duncan’s multiple range test (*p* < 0.05) (*n* = 10).

The majority of viruses within the family *Virgaviridae* (excluding genus *Tobamovirus*) encode cysteine-rich proteins (CRPs) [1,31,32]. BSMV γb protein consists of 11 cysteines, and mutations of the cysteines at positions 60, 64, 71, and 81 as well as a histidine at position 85 in the C2 region (aa 60–85) of γb, result in the loss of its zinc-binding activity [40]. Although CRPs from the genera *Hordei-*, *Peclu-*, *Gora-*, *Furo-*, and *Tobravirus* have low sequence similarity, the CCCH-type zinc-binding motifs (Cys-50, Cys-60, Cys-81, and His-85) are highly conserved (Figs 4A and S4). The alanine substitution at His-85 of γb (γb_H85A_) mutant, which impairs the CCCH motif, failed to support its interaction with CP in Y2H assay (Fig 4C), confirming the important role of CCCH motif in the CP–γb interaction. Upon abolishing the Zn^2+^-binding activity of γb by adding 200 μM EDTA, the interaction between γb and CP was reduced by about fivefold; however, the γb_H85A_ mutant showed the same weak band as γb in the EDTA-treated samples (Fig 4D). These results suggest that the interaction between γb and CP can be enhanced in the presence of Zn^2+^.

To determine whether CP has zinc-binding activity, the amino acid sequences of CP from different viruses were aligned via the Uniprot website (https://www.uniprot.org/), and the potential zinc-binding sites were predicted by using the ZincBinder online server (http://www.proteininformatics.org/mkumar/znbinder) (S5 Fig). The results showed that the histidine at position 13 (His-13) of CP is a potential zinc binding residue among hordei-, peclu-, and goraviruses in the family *Virgaviridae* (Figs 4B and S6). To investigate the spatial distribution of CP His-13 on virus particles, three-dimensional models of BSMV virions were built based on the reported near-atomic structure of BSMV [33], and the result showed that CP His-13 was located on the virion surface (S7 Fig), supporting the prediction that His-13 is critical for CP to bind Zn^2+^. Next, to investigate whether CP His-13 is critical for the interaction between γb and CP, GST pull-down was performed by using CP_H13A_, and the results showed that GST–γb barely pulled down CP_H13A_–His compared to CP–His. As expected, when the redundant EDTA was present, the interaction of γb with CP was reduced to a similar extent compared to CP_H13A_ (Fig 4E). Altogether, these results indicate the important role of Zn^2+^ in the CP–γb interaction.

Based on these results, we hypothesized that cysteine-rich γb protein might bind to rod-shaped BSMV virions with the assistance of Zn^2+^. To this end, γb His-85 and CP His-13 were substituted with alanine in BSMV infectious cDNA clone to generate BSMV_H85A_ and BSMV_CP-H13A_ mutants, respectively. Immunogold labeling experiments showed that only a few gold particles were detected on the surface of purified BSMV_H85A_ or BSMV_CP-H13A_ virions compared to the wild-type BSMV (Fig 4F and 4G). On TEM observation, the number of virions per visual field in BSMV_H85A_ and BSMV_mγb_ mutants was much less than in wild-type BSMV. To evaluate the effects of γb on the stability of BSMV virions, γb-3xFlag protein and γb_H85A_-3xFlag were transiently overexpressed in BSMV_mγb_-infected *N*. *benthamiana*, and the virions of different BSMV derivatives were purified. The TEM results demonstrated that γb, but not γb_H85A_, could restore the number of BSMV_mγb_ virions to the wild-type level (Fig 4H). Collectively, these results indicate that γb physically associates with virions by interacting with CP in a Zn^2+^-dependent manner.

### γb plays a role in BSMV virions assembly

**Fig 5:**
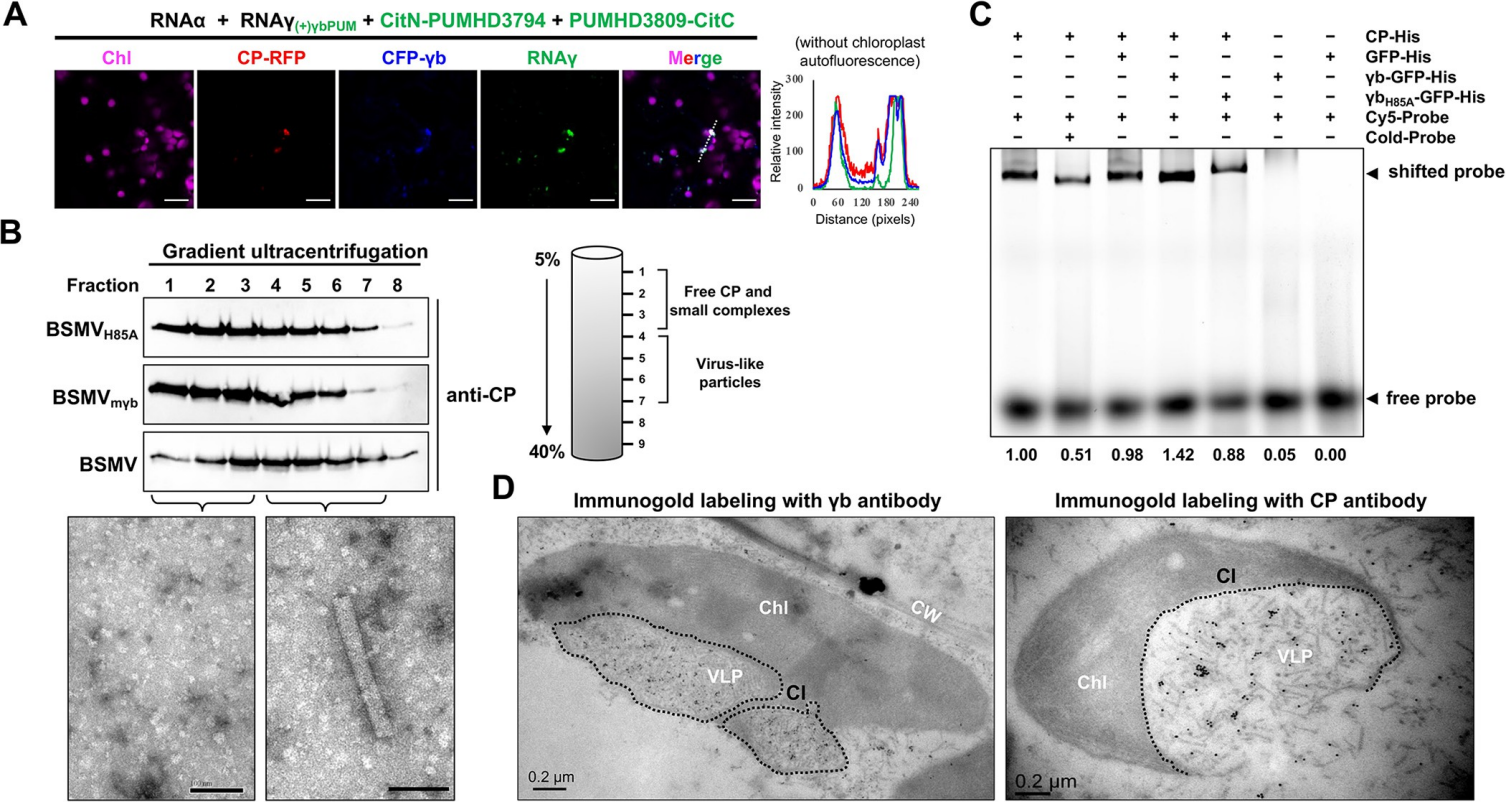
γb enhances virion assembly by interacting with CP. **(A)** Co-localization analyses of γb, CP, and plus-strand BSMV RNAs in RNAα + RNAγ- infected *N*. *benthamiana* epidermal cells at 3 dpi. Mixtures of *A*. *tumefaciens* containing CP-RFP, CFP-γb, and the split YFP-tagged Pumilio proteins were agroinfiltrated into RNAα and RNAγ_(+)PUM_-infected leaves [34]. Co-localization analyses were visualized by confocal microscopy at 3 dpi. Chloroplast autofluorescence was shown as false pink color. Figures on the right indicate the normalized fluorescence intensity of GFP, RFP, and CFP channels along the white dashed line in the merged confocal images. Scale bars, 30 μm. **(B)** Extracts of BSMV-, BSMV_mγb_-, and BSMV_H85A_- infected leaves were analyzed after sucrose gradient centrifugation. Ten fractions were collected from the bottom and the top eight fractions were subjected to CP-specific immunoblot analysis. The right panel indicates a schematic representation of VLPs separation by sucrose gradient centrifugation. The bottom panel indicates the fractions observed under electron microscope. Scale bars, 100 μm. **(C)** EMSA assay to analyze the effect of γb on the binding between CP and viral RNAs. The 300 bp RNA probe labeled with andy-fluor-647. Unlabeled RNA probe (cold probe) was used as a control. The fusion proteins and probes used for EMSA analyses are indicated on the right of each panel. **(D)** Immunogold labeling of γb and CP in BSMV-infected *N*. *benthamiana* cells show that γb protein binds to VLPs in BSMV-infected leaves. Chl, Chloroplast; CI, cytoplasmic invagination; VLP, virion-like particle. Scale bars, 0.2 μm.

γb serves as a replication enhancer [34] and facilitates the assembly of the viral movement complex at chloroplasts [35]. However, where the BSMV virions assembly occurs remains an open question. CP-GFP alone resulted in fluorescent puncta mainly present in the cytoplasm (S8A Fig), whereas CP-GFP and γb-RFP were co-localized at the periphery of chloroplasts in a movement-deficient BSMV mutant (RNAα + RNAγ)-infected *N*. *benthamiana*, which is consistent with the BiFC results in Fig 2A (S8B Fig). Furthermore, to investigate whether the localization of CP–γb complexes correlates with BSMV genomic RNA, CP-GFP and γb-RFP were co-expressed with the plus-sense BSMV Pumilio system [34]. The results revealed that CP-GFP and γb-RFP were co-localized with BSMV genomic RNA at the periphery of chloroplasts (Fig 5A), implying that chloroplasts may be the location where the assembly of BSMV virions occurs.

To further determine whether γb participates in virion assembly, BSMV, BSMV_mγb_, and BSMV_H85A_ were agroinfiltrated into *N*. *benthamiana* leaves, and native extracts were subjected to sucrose gradient centrifugation at 4 days post-infiltration (dpi). Consistent with previous studies [15], the top layer (fractions 1 to 3) were free CP and small-volume CP complexes, while the middle layer (fractions 4 to 7) corresponded to BSMV VLPs, which was further confirmed by TEM (Fig 5B). The results show a substantially higher proportion of CP proteins in the middle layer in BSMV-infected samples. However, in the absence of γb protein expression (BSMVmγb) or when the γb-CP interaction was disrupted in the BSMV_H85A_ mutant, the majority of CP proteins were detected in the top layer (Fig 5B). Altogether, these results suggest that γb promotes the formation of BSMV VLPs.

Given the apparent co-localization of γb and CP in close proximity to the chloroplasts which are the major sites for BSMV replication and contain abundant viral progeny RNAs [34], to determine whether γb promotes BSMV virion assembly by enhancing the RNA-binding activity of CP, we performed an EMSA assay. Since the tripartite genomic RNAs of BSMV shared a highly conserved 3’-UTR, the 300 bp RNA at 3’-UTR labeled with Andy Fluor-647 was used as probe [31,34,35]. A specific band appeared when the CP-His protein was included, indicating that CP specifically binds to this RNA probe (Fig 5C). Intriguingly, γb, but not γb_H85A_, could significantly enhance CP RNA-binding activity (Fig 5C). Taken together, these results show that γb protein is required for virion assembly by promoting CP RNA-binding capacity.

Previous study has shown that BSMV infection induce cytoplasmic invaginations (CIs) on the chloroplast in BSMV-infected *N*. *benthamiana* leaf cells [41]. To further observe virion assembly in BSMV-infected cells, the agroinfiltrated *N*. *benthamiana* leaves were harvested at 3 dpi, followed by chemical fixing and embedding in resin for immunogold labeling analysis. The results reveal that large quantities of VLPs in CIs of the abnormal chloroplast were labeled with gold particles conjugated to CP antibodies in BSMV-infected leaves. Moreover, gold particles conjugated to γb protein also bound specifically to the surface of these VLPs *in vivo* (Fig 5D). These results indicate that γb protein tightly binds to BSMV virions *in vivo* and may coordinate encapsidation at virion assembly sites. Beyond that, we provide persuasive evidence that beyond BSMV replication sites, BSMV virion assembly also occurs at chloroplasts. The close link between BSMV replication and encapsidation is essential for facilitating effective plant infection.

### γb with zinc-binding activity is required for the stability of BSMV virions

**Fig 6:**
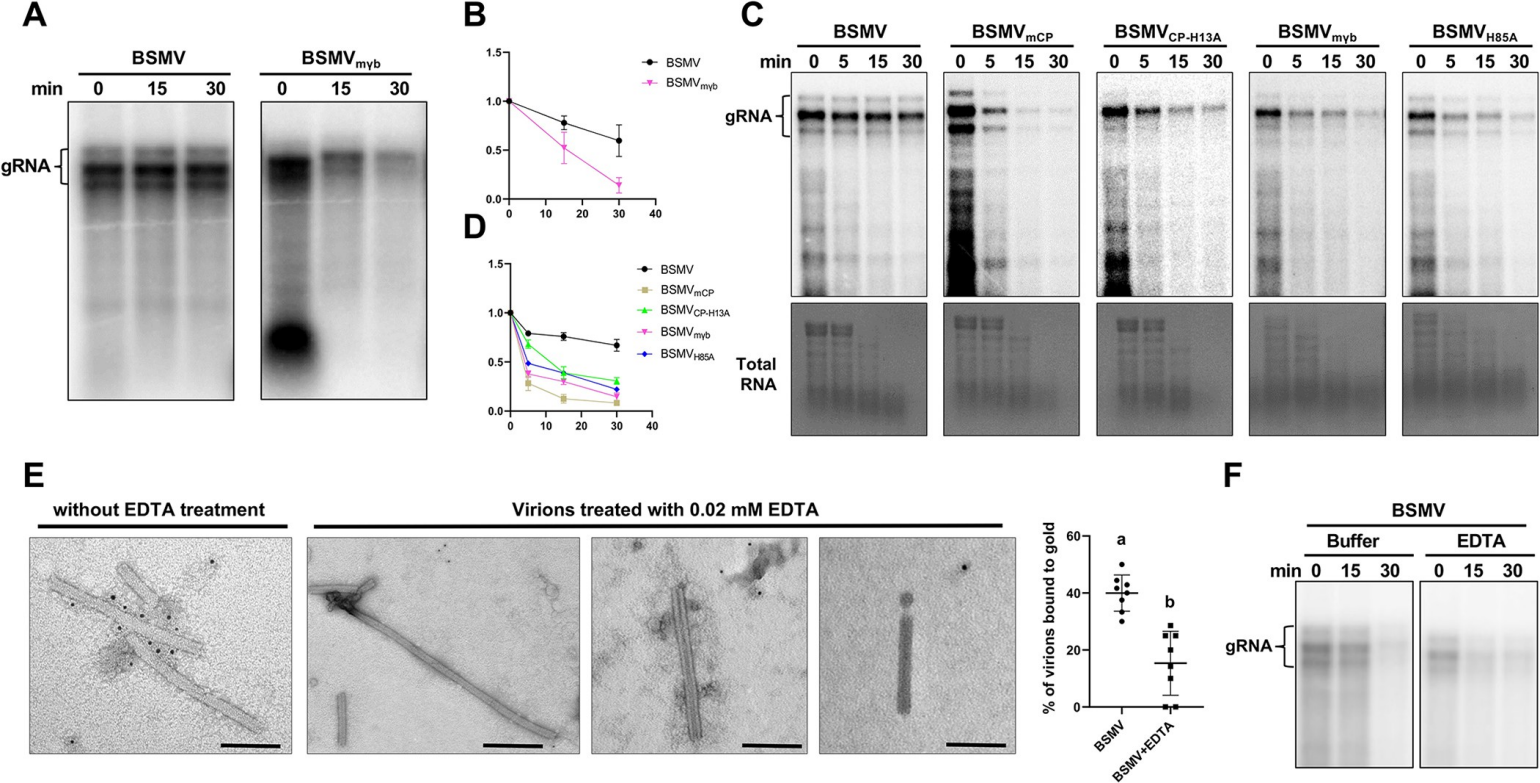
γb is required for the stability of BSMV virions. **(A)** RNase sensitivity assay in BSMV and BSMV_mγb_ virions extracts. 0.2 ng/μL RNaseA were added to the purified BSMV and BSMV_mγb_ virions, and incubated at 37°C at 0 to 30 min. Viral RNA was extracted followed by Northern blot. **(B)** Quantification of viral RNA degradation rates in Fig 6A. The relative viral RNA level at 0 min was set to 1.0. Data are shown from three independent repeats; error bars indicate standard deviation (*n* = 3). **(C)** Endogenous RNase sensitivity assay in extracts from *N*. *benthamiana* infected with BSMV and its derivatives. Inoculated leaves were ground and incubated in PIPES buffer at 37°C at 0 to 30 min, to allow degradation of unprotected RNA by the endogenous RNase. The total RNAs were then extracted and analyzed by Northern blot. Methylene blue-stained rRNAs served as RNA loading controls. **(D)** Quantification of viral RNA degradation rates in Fig 6C. The relative viral RNA level at 0 min was set to 1.0. Data are shown from three independent repeats; error bars indicate standard deviation (*n* = 3). **(E)** Immunogold labeling experiments show that Zn^2+^ can enhance virion stability by promoting γb binding to virions. The purified BSMV virions were treated with 0.02 mM EDTA at 4°C for 12 h, followed by immunogold labeling assays. The first column (without EDTA treatment) serves as negative control. Average amounts of γb protein that associated with purified BSMV virions with or without EDTA treatment as shown in the right panel. Different letters above the bars denote statistically significant differences (*p* < 0.05) determined by the Duncan’s multiple range test (*n* = 8). Scale bars, 100 μm. **(F)** RNase sensitivity assay in extracts of BSMV virions under the EDTA treatment. The purified BSMV virions were treated with 0.02 mM EDTA or PBS buffer and incubated with 1 nM RNaseA at 37°C at 0 to 30 min. Viral RNA was extracted and detected by Northern blot.

Preliminary data suggest that γb may also stabilize BSMV viral particles, which could play dual roles in BSMV morphogenesis (Fig 4H). To clarify this, purified virions were incubated with 1 nM RNaseA at 37°C, and the result shows that the degradation rate of BSMV_mγb_ genomic RNAs was significantly higher than that of the BSMV (Fig 6A and 6B). Next, *Agrobacteria* containing BSMV, BSMV_mCP_, BSMV_CP-H13A_, BSMV_mγb_, or BSMV_H85A_ infectious clone were individually infiltrated into *N*. *benthamiana*, infiltrated leaf tissues were harvested at 4 dpi and ground in liquid nitrogen, crude extracts were incubated at 37°C followed by *in vivo* endogenous RNase sensitivity assay [42, 43]. Northern blot results show that BSMV genomic RNA was quite stable, owing to the protection by intact particles. However, the viral genome of BSMV_CP-H13A_ and BSMV_H85A_ were degraded quickly, consistent with BSMV_mγb_ and BSMV_mCP_ mutants (Fig 6C and 6D). These results indicate that γb protein can stabilize BSMV particles *in vivo* and *in vitro*, coordinating with CP to encapsulate the viral genome and protect it from degradation.

Considering that Zn^2+^ tightens the association of γb protein with BSMV virions, we wondered whether Zn^2+^ could enhance virion stability by promoting γb binding to virions. To test this, purified BSMV virions were treated with 0.02 mM EDTA, followed by immunogold-labeling assays with antibodies against the γb protein. The results show that the number of gold particles labeled on the surface of BSMV virus particles was significantly reduced under EDTA treatment (Fig 6E), and the virion morphology also changed (Fig 6E). To further confirm these results, RNase sensitivity assay was carried out. Purified BSMV virions were incubated in PBS buffer with 0.02 mM EDTA for 12 h, followed by 1 nM RNaseA treatment. The result shows that the EDTA treatment significantly decreased the stability of BSMV virions compared to PBS control (Fig 6F). Taken together, these results indicate that stability of BSMV virions requires the association of γb protein with virions.

### Physical association of the CRPs with the virions is a general feature in rod-shaped viruses

Our data demonstrate that γb is associated with the surface of BSMV virions in a Zn^2+^-dependent manner and is involved in virion assembly and stability. Moreover, since the sequence alignment indicated that zinc-binding amino acids are highly conserved in CRPs and CPs of diverse viruses (Figs 4A and 4B and S4 and S6), we wondered whether the physical association of CRPs with virions is a general feature in rod-shaped plant viruses. To test this, LRSV, PSLV, and TRV in the family *Virgaviridae* [1], and beet necrotic yellow vein virus (BNYVV; genus *Benyvirus*) in the family *Benyviridae* [2] were used for further analysis. BiFC assay confirmed the interaction between CRPs and CPs (S9 Fig), indicating that these CRPs also associate with their corresponding CPs.

**Fig 7:**
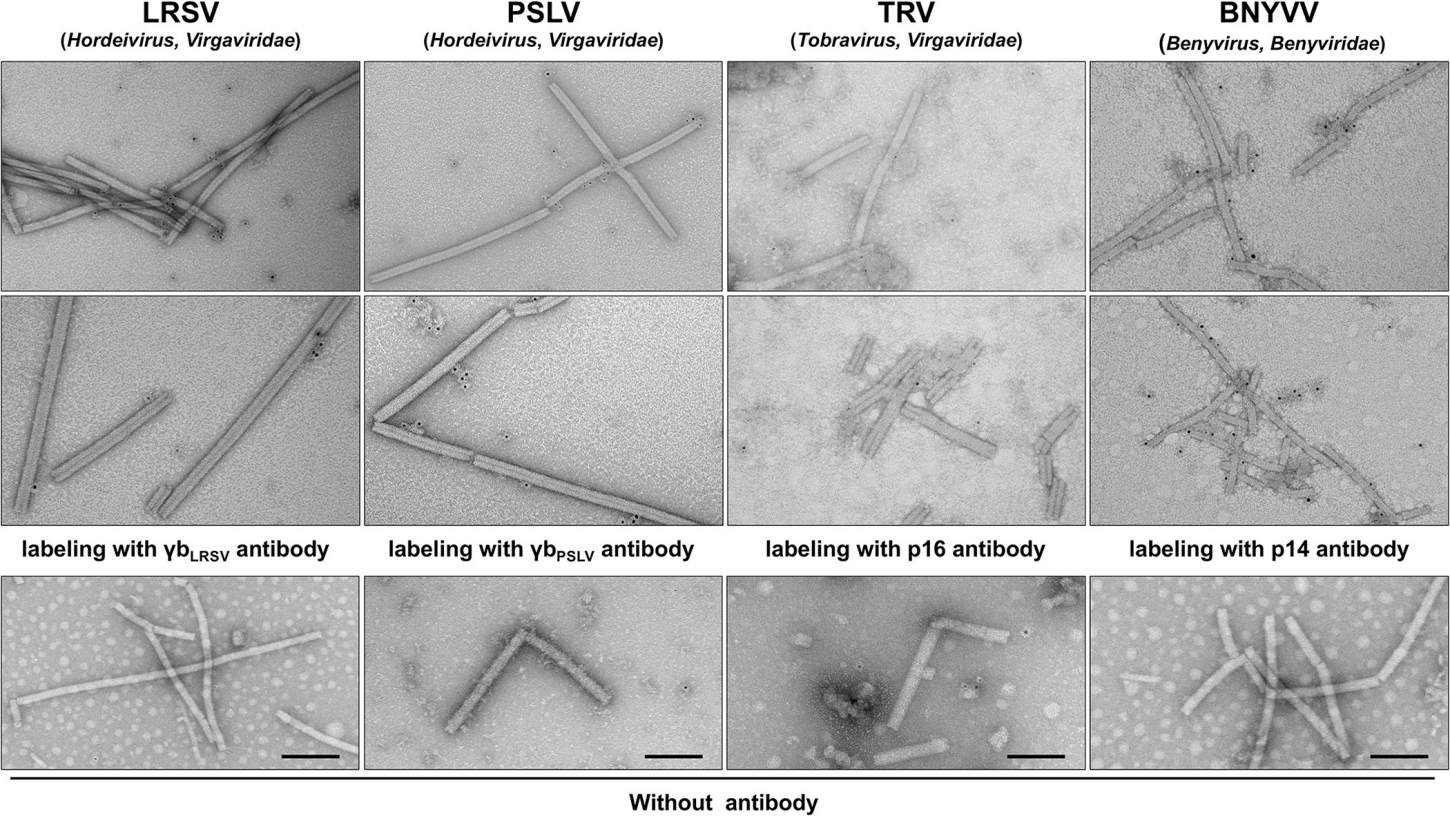
The physical binding of CRPs to the corresponding virions is a general feature among rod-shaped viruses. The virions of LRSV, TRV, and BNYVV were purified from the infected *N*. *benthamiana* leaves, the virions of PSLV were purified from infected barley leaves (Yangfu 4056) at 10 dpi. The virions were adsorbed onto 200-mesh nickel grids, followed by incubated with antibodies against the corresponding CRPs or BL buffer (as control) at room temperature for 2 h. The pictures were visualized by TEM at 80 kV. Scale bars, 100 μm.

To further investigate the relationship between CRPs and CPs, we performed immunogold-labeling assays. The purified LRSV [44], PSLV [45], TRV [13,46], and BNYVV [47] virions were adsorbed onto Formvar/carbon-coated nickel grids, followed by incubation with the corresponding CRP antibodies, and the samples were observed by TEM. The results show that LRSV γb, PSLV γb, TRV p16, and BNYVV p14 all tended to bind to their respective virions with different binding affinities (Fig 7). Taken together, these data reveal a new role of CRPs on virions, which might play important roles in virus morphogenesis among different rod-shaped viruses.

## Discussion

The life cycle of plant viruses includes viral replication, intra- and intercellular movement, and encapsidation; not only the movement, but also the encapsidation are also coupled with viral replication [48–50]. Most viruses contain small genomes due to the limitation imposed by encapsidation; in order to efficiently infect host plants, viral proteins interact with each other, resulting in a combinatorial interaction network [51–53]. The CP protein plays a dominant role in the encapsidation process of viral particles, but little is known about how other viral proteins function in virion assembly. We previously demonstrated the important role of multifunctional γb protein in viral replication [34], cell-to-cell movement [35], and replication-to-movement switch [37]. In this study, we extended our investigation by demonstrating that γb binds to virions via physical interaction with CP in a Zn^2+^-dependent manner, which is required for virion assembly and stability. These data reveal a mechanism whereby the multifunctional CRP protein γb directly participates in BSMV virion assembly, which revealed a novel role of γb protein beyond its functions in multiple steps of viral infection, including replication, movement, and assembly.

Self-assembly of virions is a complex oligomerization process, which usually occurs along ordered interactions between CP subunits and includes a series of transient assembly intermediates [7]. Once assembled, most virions undergo maturation reactions, such as covalent modification or conformational rearrangement, to increase their stability and protect the viral genome from physicochemical attack [54]. However, the virion also has a metastable and dynamic structure that undergoes controlled conformational transitions during viral infection and performs critical functions [7,54–56]. Unlike other helical plant viruses, BSMV has two versions of virions, one wider and one narrower. The narrower version contains two lateral inter-subunit salt bridges and is more stable, while the wider version has no inter-subunit salt bridges [57]. Here, we found that the binding of γb to virions enhanced the assembly and stability of BSMV virions. Whether the γb–CP interaction alters the inter-subunit contact of the CP subunit and mediates the transition from a wider to a narrower version is unclear, and crystal structural evidence of the γb–CP complex may be helpful to address this question.

An increasing body of evidence indicates that viral-encoded multifunctional small proteins and host factors play important roles in the assembly and disassembly of virions. For example, HC-Pro is associated with PPV particles at one end of the virion to enhance the stability and yield of infectious PPV particles [16,58,59]. TGB1 has a negative effect on PVX particles by associating with one end of the particles to mediate their disassemble process [17]. In addition, host factor Hsc70-2 is physically bound to CNV and necroviruses and is involved in the stability of virions and viral systemic movement [23,43,60]. In this study, we found that γb proteins are attached to the surface of rod-shaped BSMV virions. Our findings are completely different from previous findings regarding the binding mode of potyviral HC-Pro [15,16], PVX TGB1 [17], and the CP readthrough proteins of PMTV [19, 20] and BNYVV [21], as they are all associated with virus particles at one end of the virion. These findings shed new light on the important role of non-coat proteins in virion assembly and stability. However, whether the association of γb and virions improves the virus performance or even seed transmission efficiency in natural conditions is still an open question.

The structural Gag protein encoded by HIV-1 can bind Zn^2+^ through two CCHC zinc finger structures and function in recognizing viral RNA to initiate virion assembly [27,28,61]; however, the function of Zn^2+^ and zinc finger motif, especially in plant viruses, remains elusive. The majority of rod-shaped viruses in the family *Virgaviridae* (excluding genus *Tobamovirus*) and *Benyviridae* [1,2], many filamentous viruses from *Allexivirus* and *Mandarivirus* (family *Alphaflexiviridae*) and *Vitivirus* and *Carlavirus* (family *Betaflexiviridae*) encode CRPs [10]. CRPs play an important role during viral infection, usually acting as viral suppressors of RNA silencing and pathogenicity determination [10–14]. Although CRPs in various genera have low amino acid sequence similarity, they can be divided into two groups based on their conserved domain and subcellular localization. CRPs encoded by hordei-, tobra-, furo-, gora-, and pecluviruses share the highly conserved CCCH-type motif (Cys-50, -60, -81, and His-85 in BSMV γb protein) (Figs 4A and S5A), and are mainly localized in the cytoplasm; on the other hand, CRPs encoded by beny-, allexi-, mandari-, viti-, and carlaviruses have a genus-specific CCCC or CCHC-type zinc finger motif (Cys-70, -73, -107, and -110 in BNYVV P14) (S4B Fig), and an arginine-rich nuclear localization signal (NLS), which is involved in nucleocytoplasmic distribution [2, 10, 14]. Given that the binding of CRPs to virions is a general characterization (Fig 7), it can be assumed CRPs that share highly conserved CCCH-type (family *Virgaviridae*) or CCCC-type (family *Benyviridae*) zinc-binding motifs may play an important role in the morphogenesis of rod-shaped plant viruses.

Intriguingly, CPs from hordei-, gora- and pecluviruses have conserved His-13 at their N-terminal and are distributed on the surface of virions, which is predicted as a zinc binding residue (S6 and S7 Figs). Benyviral CP also contains a His-15 that may be involved in zinc binding activity (S10 Fig). Interestingly, we found that there was a conserved histidine at the C-terminus of tobraviral CP, and the furoviral CP contained a possible HHCC-type zinc finger motif; pomovirus has two histidines around His-55 and His-140, which may be potential binding sites for zinc ions. In summary, both CPs and CRPs of almost all members of the families *Virgaviridae* and *Benyviridae* have potential Zn^2+^ binding sites (S10 Fig), thus targeting the CRPs to virions may be a general strategy employed by diverse rod-shaped and filamentous plant viruses for virus morphogenesis.

## Materials and methods

### Plant growth conditions

Growth conditions of *N*. *benthamiana* plants and barley (Yangfu 4056) were described previously [37].

### Plasmid construction

All constructs described below were validated by DNA sequencing. The corresponding primers used in this study are listed in S2 Table.

The BSMV (ND18 strain) infectious cDNA clones have been described previously [62]. CP_H13A_ and γb_H85A_ derivatives in pCB301-RNAβ and pCB301-RNAγ were constructed by using reverse-PCR approach.

For GST pull-down assays, BSMV γb and its derivatives were individually cloned into pGEX-KG vector [63], BSMV CP and its derivatives were cloned into pET30a (+) vector. For MST assay, BSMV γb and its derivatives were individually cloned into pET30a-GFP vector. All the recombinant proteins were purified by using *Escherichia coli* (BL21 strain).

For BiFC assay, various CP and γb derivatives engineered into pSPYNE-35S and pSPYCE-35S split YFP destination vectors which have been described previously [64].

For subcellular localization analyses, BSMV CP was cloned into pGDGm vector [65]. γb and its derivatives were cloned into pGDRm and pGD-CFP vector at *Sal*I sites [65], respectively.

For co-immunoprecipitation (co-IP) experiments, γb and its derivatives were integrated into *Spe*I and *Sac*I restriction sites of pMDC32 vector [43].

### Virus inoculation

For *N*. *benthamiana* leaves, *Agrobacterium tumefaciens* EHA105 containing pCB301-RNAα, pCB301-RNAβ, and pCB301-RNAγ (or its derivatives) were mixed at equal volumes to a final OD_600_ = 0.3, and infiltrated into leaves of 3-4-week-old *N*. *benthamiana* plants. For barley leaves, *N*. *benthamiana* leaves inoculated with BSMV or its derivatives were harvested at 5–10 dpi and then ground in 10 mM sodium phosphate (pH 7.2) containing 0.5% freshly prepared sodium sulfite, and barley leaves at the 2-leaf stage are inoculated directly with the ground sap of *N*. *benthamiana* leaves by mechanical rubbing [66,67].

### Virion purification and VLPs isolation

Viral inoculated leaves were collected at 5–10 dpi and ground in liquid nitrogen. 10 g *N*. *benthamiana* leaf tissue was extracted in 20 mL precooled 0.5 M boric acid buffer (pH 9.0). After filtering with gauze, the homogenate was centrifuged at 8000 *g* for 10 min at 4°C. 1/20 volume of the supernatant 20% Triton X-100 was added into the crude extract, and then subjected to ultracentrifugation at 40,000 rpm for 2 h at 4°C in a Hitachi type P70AT rotor. The pelleted was resuspended in 1.5 mL 50 mM PB (0.1 M KH_2_PO_4_, 0.1M K_2_HPO_4_, pH 6.85), and then add 20% Triton X-100 into a final concentration of 1%, which is subjected to sucrose density gradient centrifugation at 35,000 rpm for 2 h at 14°C in a Hitachi type P40ST rotor. The pellet was resuspended in 1 mL 10 mM PB, and the supernatant were BSMV virions. The virus concentration was determined spectrophotometrically, the absorbance at 260 nm of a 1 mg/mL suspension of BSMV is 2.6.

Isolation of BSMV VLPs was performed as described previously with minor modifications [15]. Briefly, infiltrated *N*. *benthamiana* leaf tissues (250–300 mg) were harvested at 4 dpi, ground in liquid nitrogen and suspended in 200 μL borate buffer. The mixtures were centrifuged at 4°C for 10 min at 3,000 *g*, and subjected to centrifugation in continuous sucrose gradients (5 to 40%) of 4.8 mL in borate buffer. The gradients were centrifuged at 4°C for 20 min at 230000 *g* in a Hitachi type SW55 rotor. Fractions of approximately 300 μL were collected by capillary gravity from the bottom of the tube via a syringe and subjected to western blot analysis.

Virion purification of LRSV [44], PSLV [45], TRV [13,46], and BNYVV [47] as described previously with minor modifications.

### Transmission electron microscopy (TEM)

TEM was performed as described previously [41]. Purified virions were adsorbed onto 200-mesh nickel grids for 5 min. The samples were stained with 2% uranyl acetate for 1 min in the dark, the grids were then viewed with a Hitachi H-7650 or a JEM-1230 transmission electron microscope operated at 80 kV.

### Immunogold labeling assay

Immunogold labeling assay was performed as described previously with minor modifications [41]. Purified virions were adsorbed onto 200-mesh nickel grids for 5 min, and dry the grids. Then placed the grids on BL buffer [1×PBST (pH 7.5, 0.05% Tween 20), 0.05% Triton X-100, and 1% BSA] for 10 min to reduce nonspecific binding of antibodies. Rabbit anti-γb polyclonal antibody was diluted 1:100 in BL buffer incubated with the nickel grids at room temperature for 2 hours. Buffer without antibody serves as negative control. The grids were washed 6 times with BL buffer for 2 min each. Dry the nickel grids under the heating lamp. The goat anti-rabbit secondary antibody conjugated with 10-nm gold particles (Sigma) was diluted as the ratio 1:100 in BL buffer and incubated for 1 hour at room temperature. After the grids were washed 6 times with BL buffer and 4 times with ddH_2_O for 2 min each, stained with 2% uranyl acetate for 1 min in the dark, dry the nickel grids again under the heating lamp. And then viewed with a Hitachi H-7650 or a JEM-1230 transmission electron microscope operated at 80 kV. The antiserums against BSMV γb and CP, LRSV γb, PSLV γb, TRV p16, and BNYVV p14 were all obtained from rabbits by Beijing Protein Innovation Co., Ltd., the pre-immune serum from rabbit was utilized in the immunogold labeling assay as a negative control.

### Microscale thermophoresis (MST)

The Microscale Thermophoresis (MST) assay was performed as described previously with minor modifications [68]. γb fused to a GFP-6×His tag was purified from *E*. *coli* BL21 strain. The concentration of the γb-GFP-His should yield between 100–1500 fluorescence counts when measured with the Monolith NT.115. After a short incubation of the target protein γb-GFP-His with CP or metal ion ligands in PBS buffer (1xPBS, 0.1% Tween 20), the samples were loaded into Monolith NT.115 Standard Treated capillaries. Measurements were made at 25°C. And then the resulting data was digitized using the Monolith NT.115 instrument.

### Subcellular localization assays and confocal microscopy

Subcellular localization assays were carried out as described previously [37]. Co-localization analysis of fluorescence were processed on the pixel-based method with the ImageJ plot profile tool [37].

### Yeast two-hybrid (Y2H) assay

The yeast two-hybrid (Y2H) assay were performed as described previously [34].Various γb derivatives were cloned into the pGBKT7 vectors, followed by transformation into the Y2HGold strain. CP derivatives were cloned into the pGADT7 vectors, followed by transformation into the Y187 strain. Mated yeasts were cultured at 30°C by shaking at 250 rpm for 20 h. The yeast cells were collected and adjusted to an OD_600_ = 1.0, followed by gradient dilutions with ddH_2_O, and 2 μL resuspended yeast cells were pipetted onto SD/-Trp-Leu plates and SD/-Trp-Leu-His-Ade plates. The yeast is cultured at 30°C about 4 days before taking photographs.

### GST pull-down assay

The GST pull-down assays were performed as described previously [34]. Approximately 5 mg of purified GST-tag protein were co-incubated with His-tag protein in 600 μL binding buffer (50 mM Tris-HCl, pH 6.8, 500 mM NaCl, 1.5% glycerol, 0.6% Triton-X 100, 0.1% Tween 20) for 3 h at 4°C. In Zn^2+^-dependent manner assays, 20 μM Cu^2+^, Ni^2+^, Mn^2+^, Mg^2+^, Ca^2+^ ions and different concentrations of Zn^2+^ or 5 μM EDTA were added to test tubes during the incubation. The beads were washed six times with binding buffer, once every ten minutes. The washed beads were boiled 10 min after resuspended in 2×SDS loading buffer. Then analyzed by Western blot with anti-GST or anti-His antibody.

### RNase sensitivity assay

The RNase sensitivity assay was performed as described previously [42]. For *in vivo* RNase sensitivity assay, 0.1 g of virus-infected leaves were ground in liquid nitrogen and incubated in 200 μL PIPES buffer (50 mM PIPES, pH 6.7, 0.1% Tween 20) at 37°C from 5 min to 30 min. Then the total RNA was extracted. As for *in vitro* RNase sensitivity assay, 1 nmol RNaseA were added to the purified BSMV virions, and incubated at 37°C. Total RNA was extracted using Trizol reagent followed detected by Northern blot.

### Northern blot

Northern blot was performed as described previously [34,37]. For detection of the BSMV genomic RNAs, total RNA (3–4 μg) was used and separated by 1.2% agarose/1.1% formaldehyde gels. And then transferred onto Hybond-N+ nylon membranes (GE Healthcare), followed by UV cross-linking and stained with methylene blue solution (0.04% methylene blue, 500 mM NaOAc). The 294 nt 3’-UTR was cloned into pSPT18 (Roche), and linearized for transcription of plus-strand RNA of BSMV probes. [γ-^32^P] UTP labeled 3’-UTR probes of BSMV were transcripted *in vitro*, and hybridized with the nylon membranes carrying viral RNA at 65°C overnight. Nylon membranes were sequentially washed with various concentrations of SSC buffer to reduce non-specific binding of the probe and then exposed to a storage phosphor screen (GE Healthcare) for 24–48 h. The resulting data were then digitized using a Typhoon 9400 PhosphorImager (GE Healthcare).

### Electrophoretic mobility shift assays

Electrophoretic mobility shift assay (EMSA) was carried out as previously described [69]. The tripartite genomic RNAs of BSMV shared a highly conserved 3’-UTR. T7 sequence was added in the forward primer to obtain a PCR product with a T7 promoter sequence incorporated in it at 5’, which used as templates for T7 RNA polymerase *in vitro* transcription of BSMV 3’-UTR. Unlabeled UTP or andy-fluor-647-UTP was used to obtain cold probe and andy-fluor-647-labeled probe respectively. To demonstrate the specificity of binding, the unlabeled probes were added in the reaction as the cold probe control. The RNA probes were incubated with various combination of test proteins for 40 min on ice. And then the protein–RNA complexes were separated on a 6% native polyacrylamide gel followed by scanning with a sapphire biomolecular imager (Azure Biosystems).

## Supporting information

S1 Fig Genome organization of BSMV and identification of the proteins associated with purified BSMV virions by LC-MS/MS. (**A**) Genome organization of BSMV. (**B**) Purified virions from BSMV-infected *N*. *benthamiana* leaves were analyzed by Q-Exactive liquid chromatography tandem mass spectrometry (LC-MS/MS). Left panel, silver staining of the purified virus particles from BSMV-infected *N*. *benthamiana* leaves, the mock-inoculated plants served as negative controls. The visible four gel bands (black arrowhead) present in the lane of purified virions but absent in the lane of negative control were cut from 12.5% SDS-PAGE gel, followed by LC-MS/MS analysis. Right panel, LC-MS/MS results from the four groups. Potential CP- associated proteins were shown in this chart and S1 Table.
(TIF)

S2 Fig Immunogold labeling assay showed that BSMV γb directly binds to BSMV virions. (**A**) Representative immunogold labeling images show that the γb protein binds to purified BSMV virions. BSMV virions incubated with the pre-immune serum were used as a negative control (right two photos). Scale bar, 200 nm. (**B**) Representative immunogold labeling images show that the γb protein cannot bind to purified PSLV, LRSV, TRV and TMV virions. Virions were adsorbed onto 200-mesh nickel grids and incubated with antibodies against the γb protein. The pictures were visualized by TEM at 80 kV. Scale bar, 200 nm.
(TIF)

S3 Fig γb interacts with CP in BiFC assay. (**A**) The independent time course BiFC assay of Fig 2A. CP-YFPn or CP-YFPc was co-expressed with BSMV_γb-YFPn_ or BSMV_γb-YFPc_. RbcL-YFPn + BSMV_γb-YFPc_ and RbcL-YFPc+ BSMV_γb-YFPn_ complementation images serve as negative controls. Chloroplast autofluorescence is shown as false red color. Scale bars, 20 μm. (**B**) The protein expression of BiFC assays in S3A Fig with anti-GFP antibodies. The bands indicated by the black arrow are denoted as CP-YFPn/YFPc, while the bands indicated by the white arrow are referred to as BSMV_γb-YFPn_ /BSMV_γb-YFPc_. Scale bar, 30 μm.
(TIF)

S4 Fig Amino acid sequence alignment of CRPs encoded by the members in the families *Virgaviridae* and *Benyviridae*. (**A**) Complete sequence alignment of CRPs in the genera *Hordeivirus*, *Pecluvirus*, *Goravirus*, *Furovirus*, and *Tobravirus* of the family *Virgaviridae*. The highly conserved CCCH-type zinc finger motif (Cys-50, Cys-60, Cys-81, and His-85 for BSMV γb protein) are highlighted in gray. Sequences were aligned with the Uniprot online server (https://www.uniprot.org/). (**B**) Complete sequence alignment of CRPs in the family *Benyviridae* (have only one genus: *Benyvirus*). The highly conserved CCCC-type zinc finger motif (Cys-70, Cys-73, Cys-107, and Cys-110 for BNYVV P14 protein) are highlighted in gray. Sequences were aligned with the Uniprot online server (https://www.uniprot.org/).
(TIF)

S5 Fig Online prediction of the zinc-binding activity of CPs in the family *Virgaviridae*. Prediction of zinc-binding activity of diverse genera CPs from *Virgaviridae* by using the ZincBinder online server (http://www.proteininformatics.org/mkumar/znbinder).
(TIF)

S6 Fig Amino acid sequence alignment of different CPs in the families *Virgaviridae* and *Benyviridae*. Complete sequence alignment of CP proteins in Fig 4B. The CPs from hordei-, gora-, peclu-, and benyviruses contain a conserved His at its N-terminal (His-13 for BSMV, His-15 for BNYVV); tobraviral CPs have a conserved His at the C-terminus; the furoviral CP contain a potential HHCC-type zinc finger motif; pomoviruses has two His around His-55 and His-140. All the conserved His are highlighted in yellow. Sequences were aligned with the Uniprot online server (https://www.uniprot.org/).
(TIF)

S7 Fig The high-resolution structure of CP subunit and BSMV virions. (**A**) The high-resolution structure of a single BSMV CP subunit at 4.1 Å obtained by cryo-electron microscopy. (**B**) The near-atomic structure of the BSMV virions at 4.1 Å obtained by cryo-EM viewed from the side. The His-13 residue of CP highlighted in red locates on the surface of the BSMV virions. The photos are generated from the Protein Data Bank (https://www.wwpdb.org/, PDB accession numbers are 5a79 and 5a7a).
(TIF)

S8 Fig Subcellular localization of CP-GFP in *N*. *benthamian*a epidermal leaf cells. (**A**) Chloroplast autofluorescence is depicted as a false red color. Figure on the right indicate the normalized fluorescence intensities of the GFP (green) or the chloroplast autofluorescence (red) channels along the dashed white lines shown in the merged images of the magnified panel. Scale bars, 20 μm. (**B**) Co-localization analyses of CP-GFP and γb-RFP in the movement-deficient BSMV mutant (RNAα + RNAγ)-infiltrated leaves at 3 dpi. Chloroplast autofluorescence is depicted as a false pink color. Figures on the right indicate the normalized fluorescence intensity of GFP and RFP channels along the white dashed line in the merged confocal images. Scale bars, 20 μm.
(TIF)

S9 Fig BiFC assays for interactions between CRPs and CP of diverse viruses in the families *Virgaviridae* and *Benyviridae*. (**A**) BiFC assays to detect interactions between CRPs and CP of diverse viruses (LRSV, PSLV, TRV, and BNYVV). The combinations were shown on the left. *N*. *benthamiana* epidermal cells were observed by confocal microscope at 3 dpi. Scale bars, 20 μm. (**B**) Western blot to detect the proteins expression in S7A Fig. YFPn-fused proteins were identified with anti-Myc antibodies and YFPc-fused proteins were identified with anti-HA antibodies.
(TIF)

S10 Fig Phylogenetic analysis of CPs in the families *Virgaviridae* and *Benyviridae*. GenBank accession numbers of different CP proteins used for the construction of the phylogenetic tree are shown in the figure and available in the National Center for Biotechnology Information (NCBI) (https://www.ncbi.nlm.nih.gov/). The conserved Histidine site is marked on the right according to sequence analysis. The phylogenetic analyses were performed with the text neighbor-joining algorithm implemented in the MEGA5 software.
(TIF)

S1 Table Viral proteins identified by LC-MS/MS in purified BSMV virions after SDS-PAGE. (DOCX)

S2 Table Primers used for plasmid constructions and RT-qPCR analysis in this work. (DOCX)
