# Supplementary figures and images for: Zn2+-dependent association of cysteine-rich protein with virion orchestrates morphogenesis of rod-shaped viruses
Source: PLoS Pathog. 2024 Jun 17;20(6):e1012311. doi: 10.1371/journal.ppat.1012311 (PMC11213338; doi:10.1371/journal.ppat.1012311)

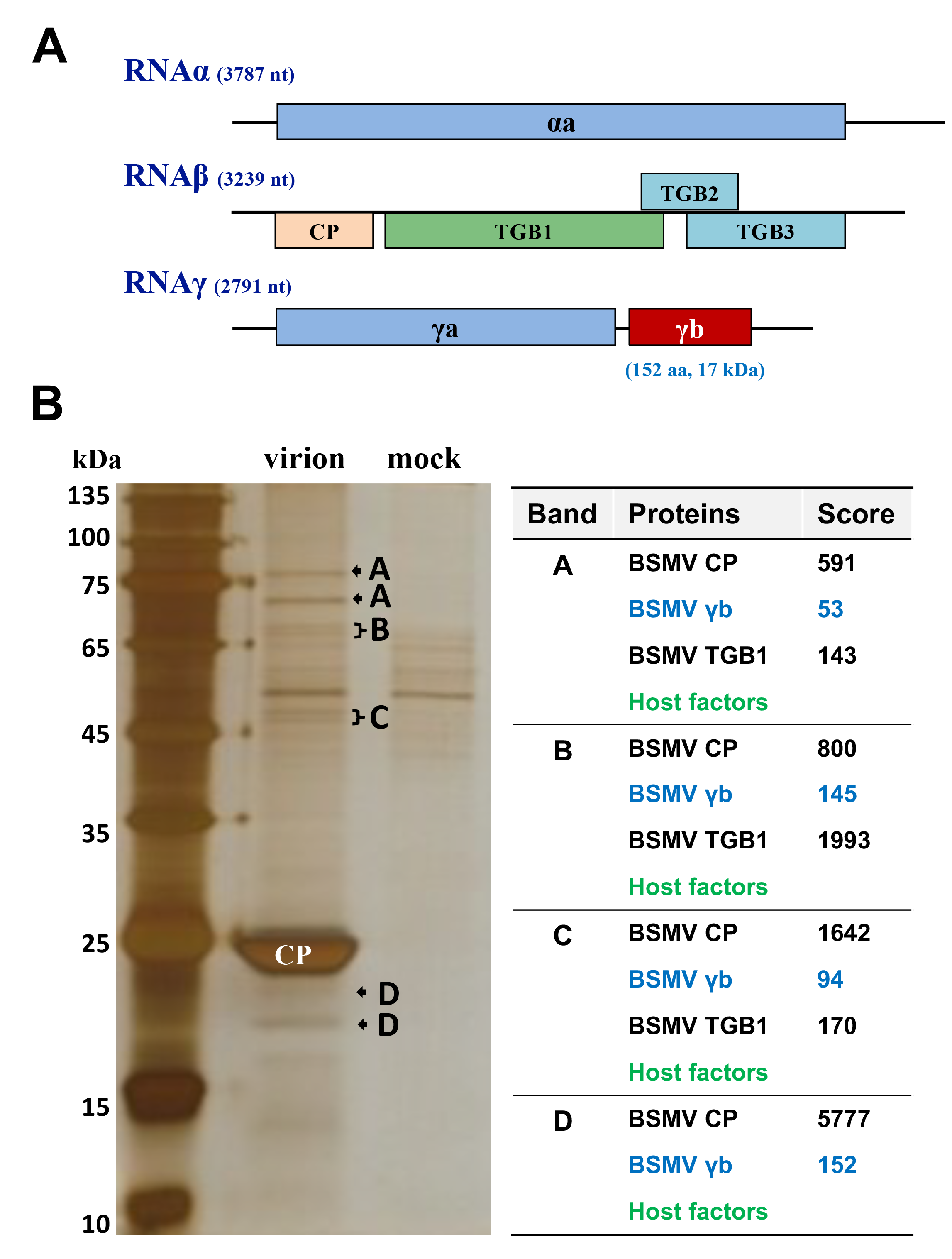

Supplement: S1 Fig — (A) Genome organization of BSMV. (B) Purified virions from BSMV-infected N. benthamiana leaves were analyzed by Q-Exactive liquid chromatography tandem mass spectrometry (LC-MS/MS). Left panel, silver staining of the purified virus particles from BSMV-infected N. benthamiana leaves, the mock-inoculated plants served as negative controls. The visible four gel bands (black arrowhead) present in the lane of purified virions but absent in the lane of negative control were cut from 12.5% SDS-PAGE gel, followed by LC-MS/MS analysis. Right panel, LC-MS/MS results from the four groups. Potential CP- associated proteins were shown in this chart and S1 Table. (TIF) [file ppat.1012311.s001.tif]

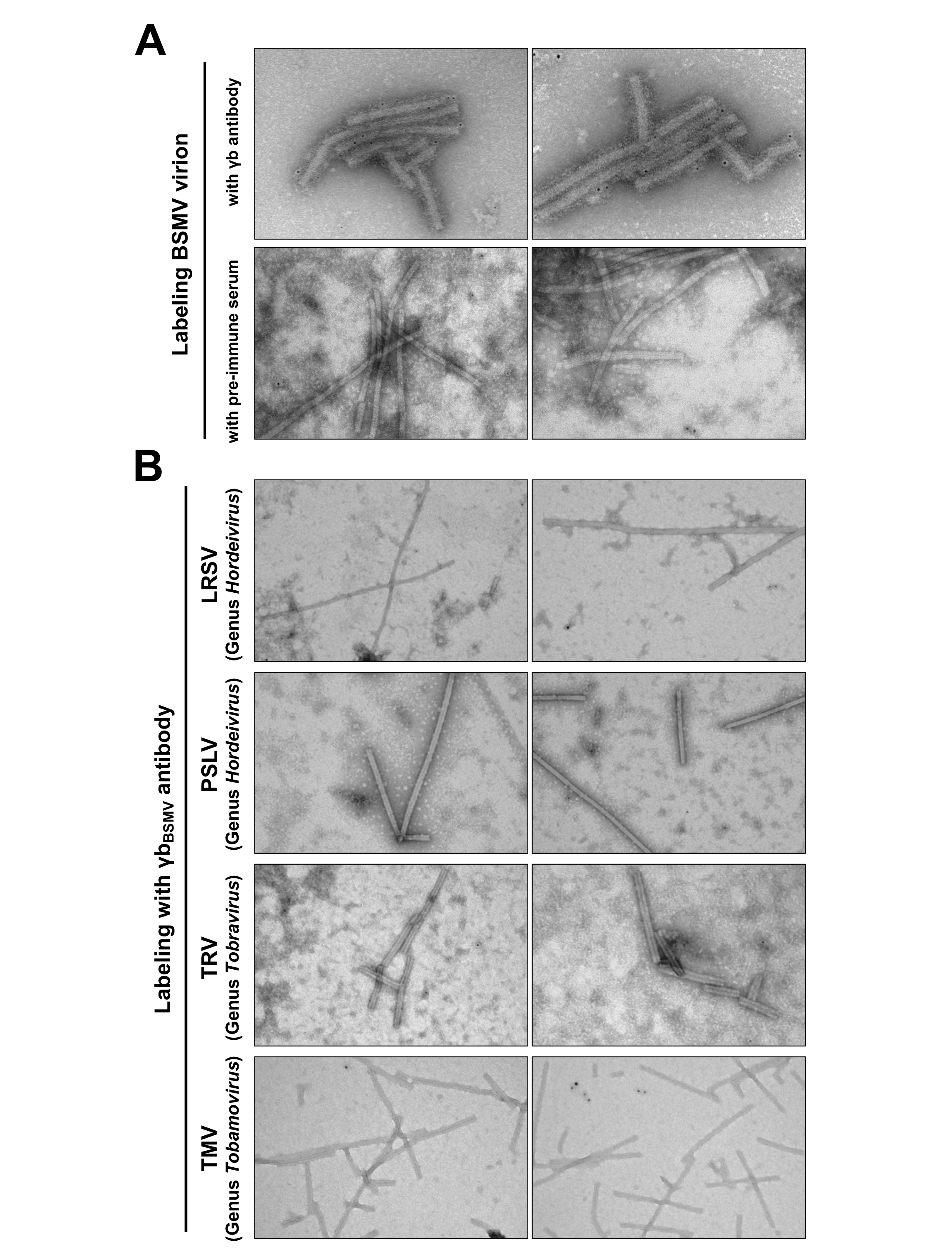

Supplement: S2 Fig — (A) Representative immunogold labeling images show that the γb protein binds to purified BSMV virions. BSMV virions incubated with the pre-immune serum were used as a negative control (right two photos). Scale bar, 200 nm. (B) Representative immunogold labeling images show that the γb protein cannot bind to purified PSLV, LRSV, TRV and TMV virions. Virions were adsorbed onto 200-mesh nickel grids and incubated with antibodies against the γb protein. The pictures were visualized by TEM at 80 kV. Scale bar, 200 nm. (TIF) [file ppat.1012311.s002.tif]

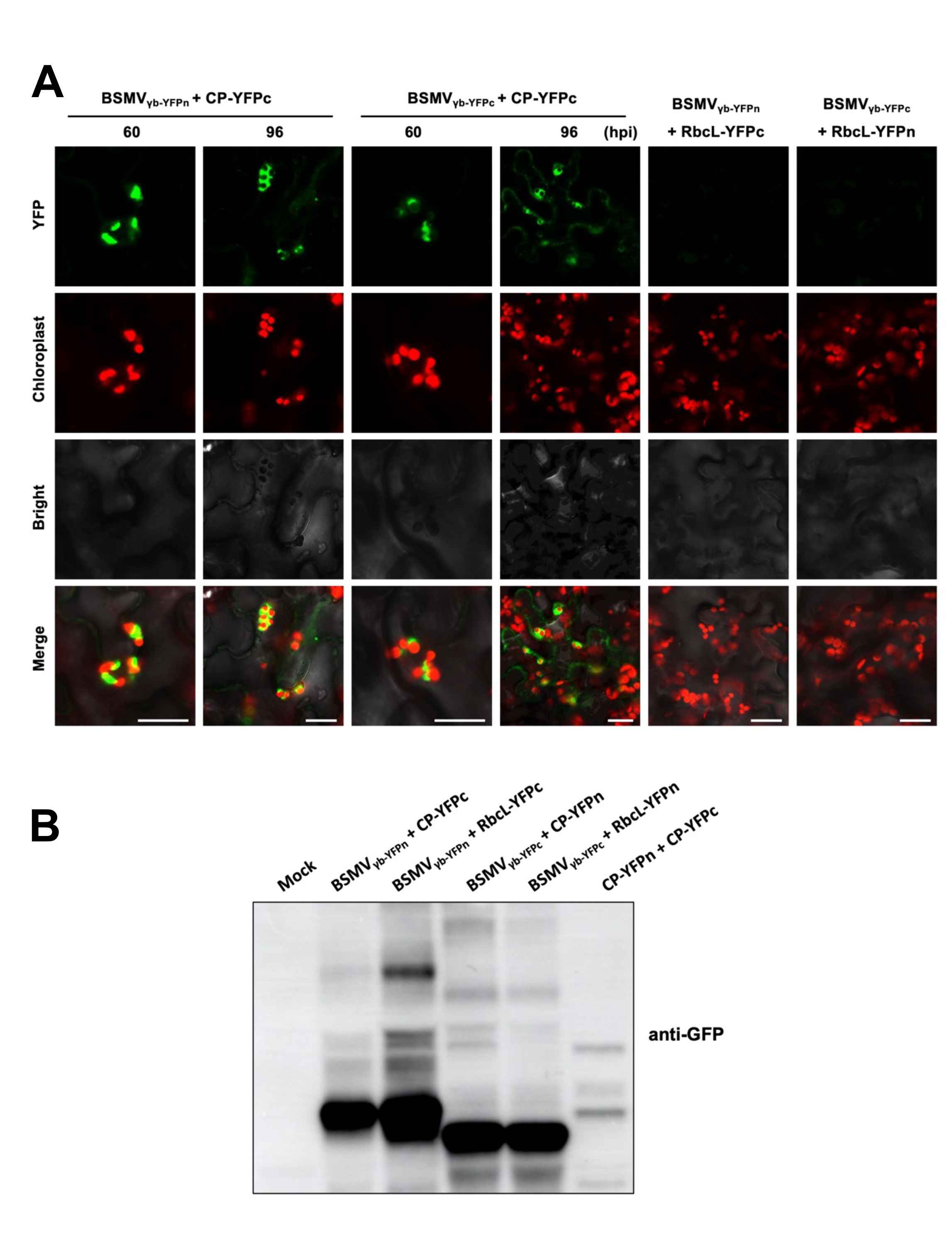

Supplement: S3 Fig — (A) The independent time course BiFC assay of Fig 2A. CP-YFPn or CP-YFPc was co-expressed with BSMVγb-YFPn or BSMVγb-YFPc. RbcL-YFPn + BSMVγb-YFPc and RbcL-YFPc+ BSMVγb-YFPn complementation images serve as negative controls. Chloroplast autofluorescence is shown as false red color. Scale bars, 20 μm. (B) The protein expression of BiFC assays in S3A Fig with anti-GFP antibodies. The bands indicated by the black arrow are denoted as CP-YFPn/YFPc, while the bands indicated by the white arrow are referred to as BSMVγb-YFPn /BSMVγb-YFPc. Scale bar, 30 μm. (TIF) [file ppat.1012311.s003.tif]

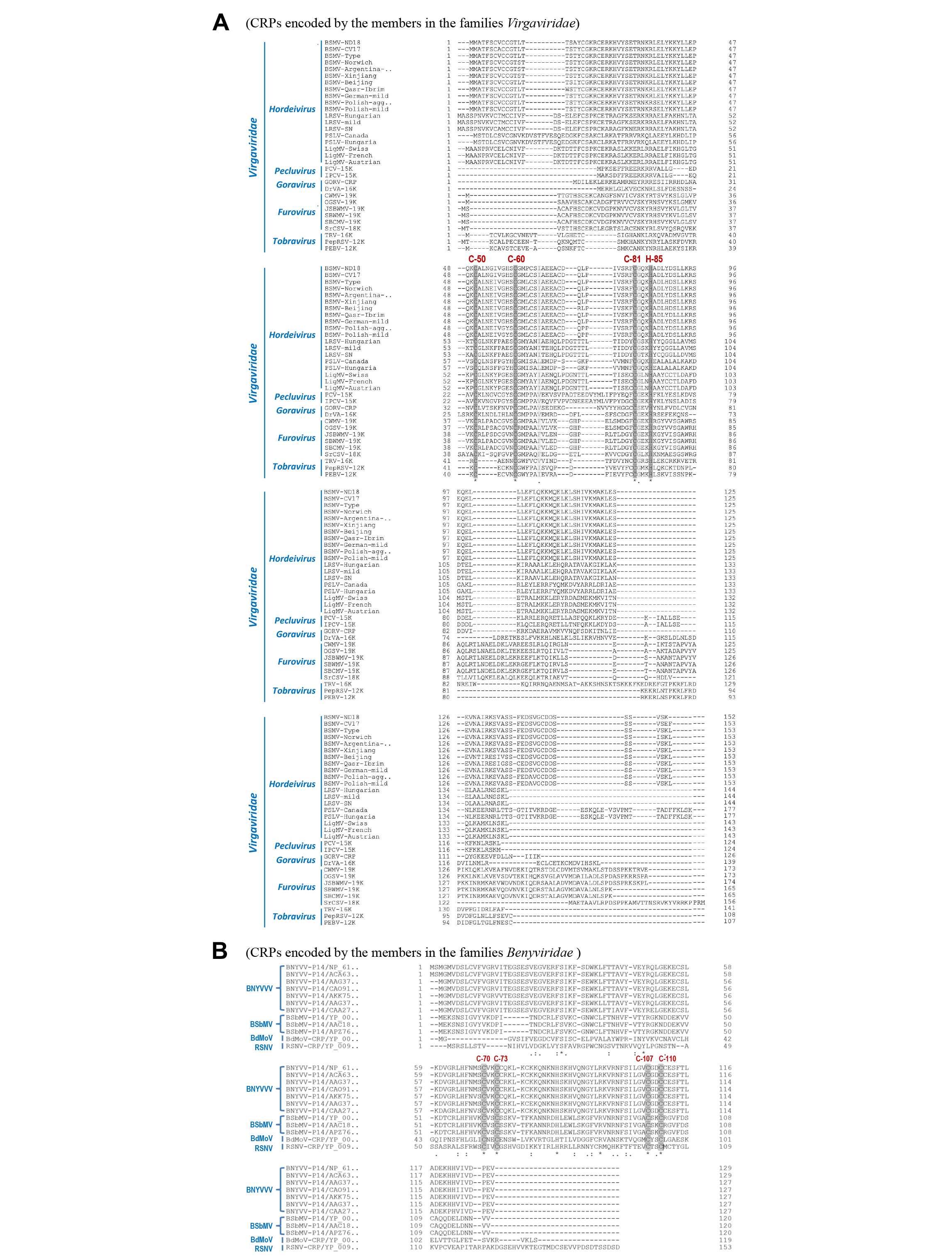

Supplement: S4 Fig — (A) Complete sequence alignment of CRPs in the genera Hordeivirus, Pecluvirus, Goravirus, Furovirus, and Tobravirus of the family Virgaviridae. The highly conserved CCCH-type zinc finger motif (Cys-50, Cys-60, Cys-81, and His-85 for BSMV γb protein) are highlighted in gray. Sequences were aligned with the Uniprot online server (https://www.uniprot.org/). (B) Complete sequence alignment of CRPs in the family Benyviridae (have only one genus: Benyvirus). The highly conserved CCCC-type zinc finger motif (Cys-70, Cys-73, Cys-107, and Cys-110 for BNYVV P14 protein) are highlighted in gray. Sequences were aligned with the Uniprot online server (https://www.uniprot.org/). (TIF) [file ppat.1012311.s004.tif]

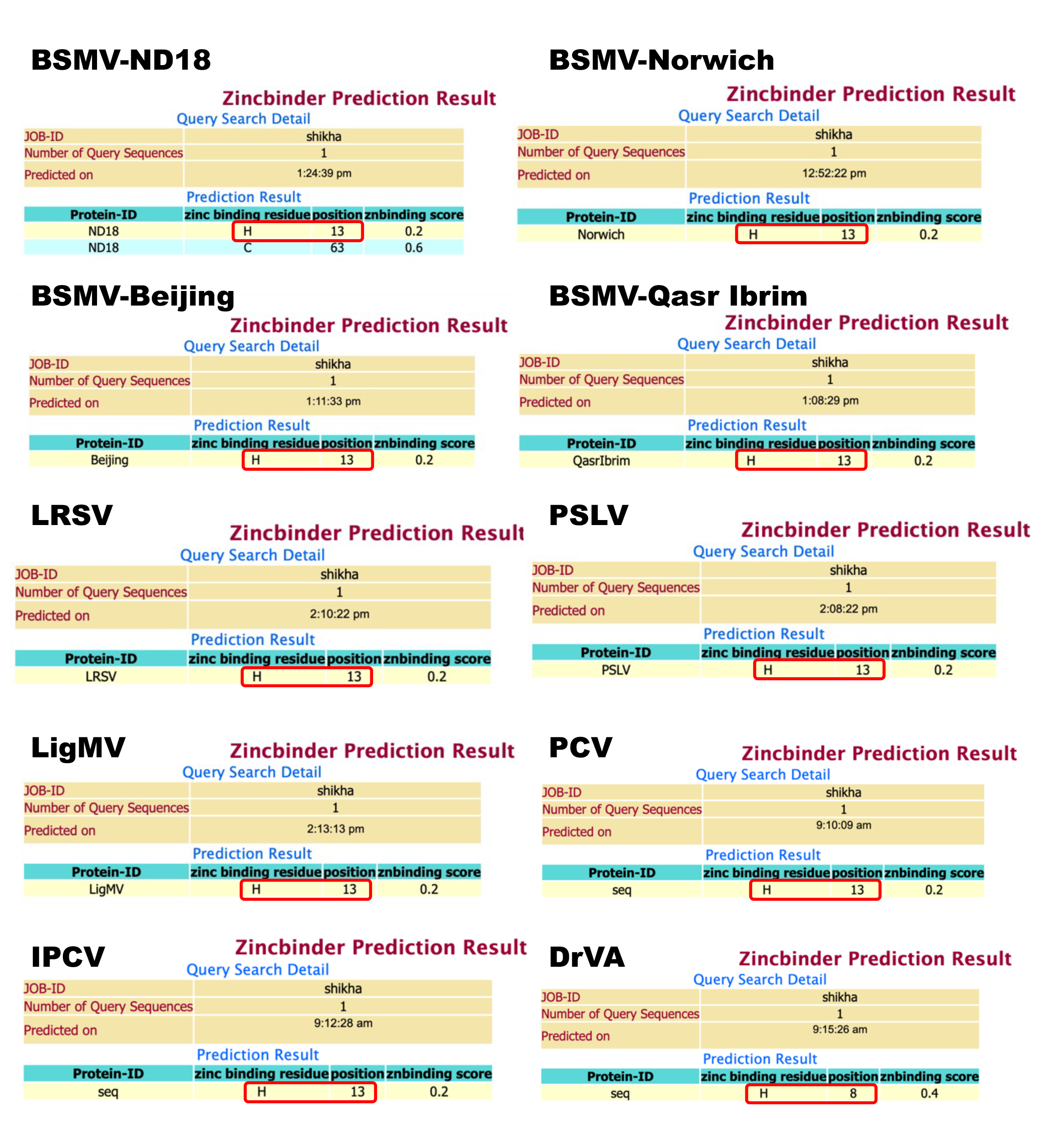

Supplement: S5 Fig — Prediction of zinc-binding activity of diverse genera CPs from Virgaviridae by using the ZincBinder online server (http://www.proteininformatics.org/mkumar/znbinder). (TIF) [file ppat.1012311.s005.tif]

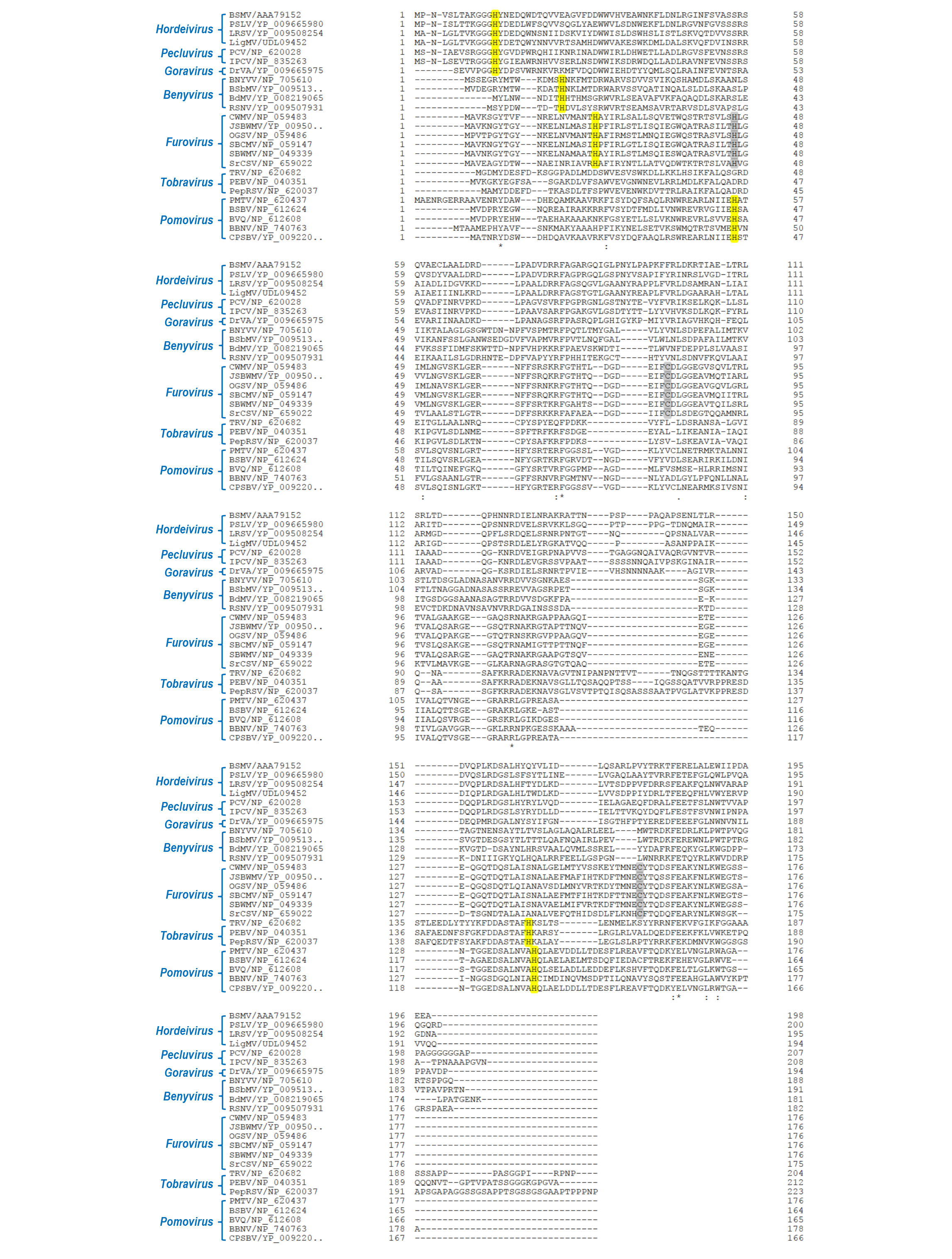

Supplement: S6 Fig — Complete sequence alignment of CP proteins in Fig 4B. The CPs from hordei-, gora-, peclu-, and benyviruses contain a conserved His at its N-terminal (His-13 for BSMV, His-15 for BNYVV); tobraviral CPs have a conserved His at the C-terminus; the furoviral CP contain a potential HHCC-type zinc finger motif; pomoviruses has two His around His-55 and His-140. All the conserved His are highlighted in yellow. Sequences were aligned with the Uniprot online server (https://www.uniprot.org/). (TIF) [file ppat.1012311.s006.tif]

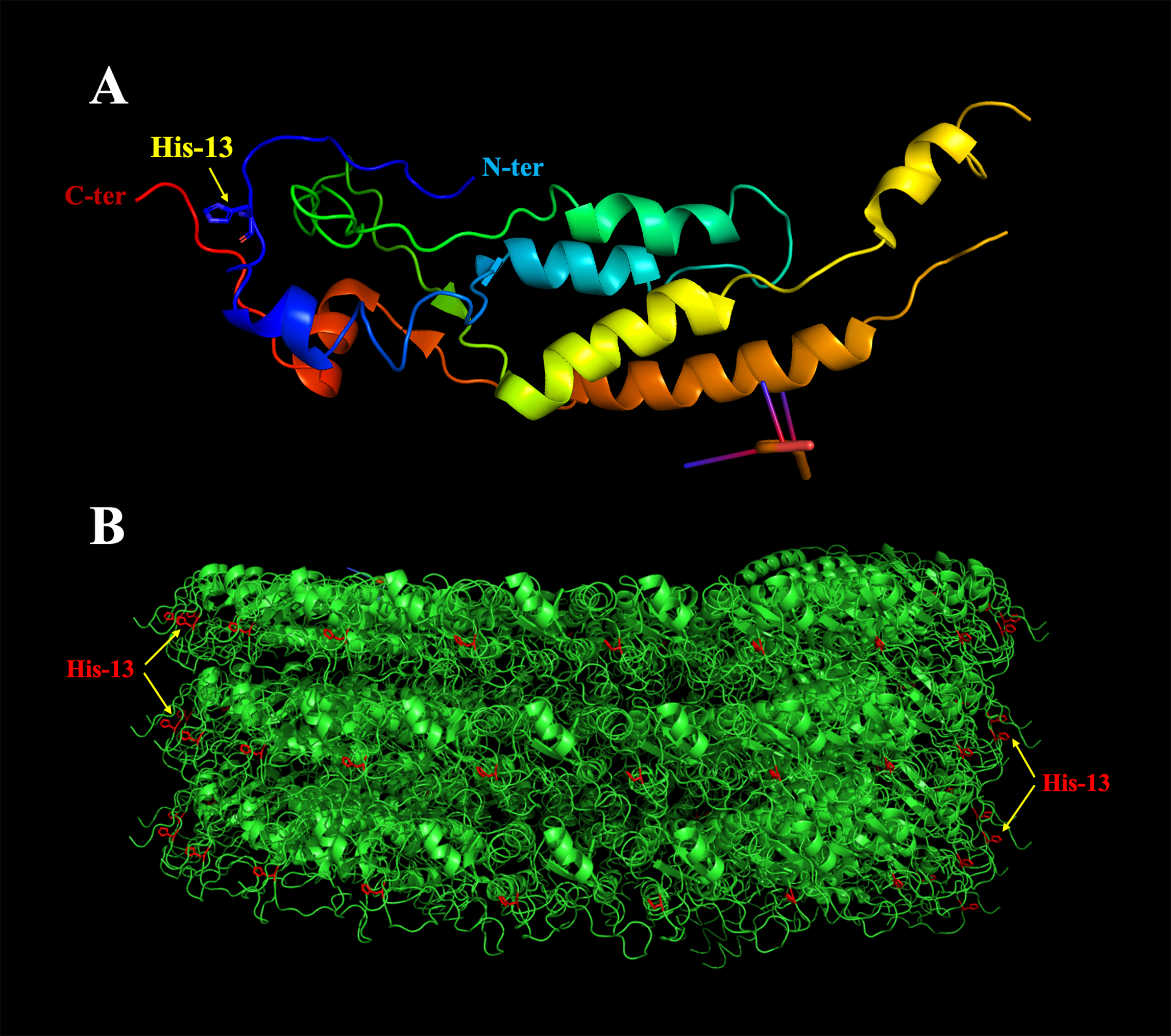

Supplement: S7 Fig — (A) The high-resolution structure of a single BSMV CP subunit at 4.1 Å obtained by cryo-electron microscopy. (B) The near-atomic structure of the BSMV virions at 4.1 Å obtained by cryo-EM viewed from the side. The His-13 residue of CP highlighted in red locates on the surface of the BSMV virions. The photos are generated from the Protein Data Bank (https://www.wwpdb.org/, PDB accession numbers are 5a79 and 5a7a). (TIF) [file ppat.1012311.s007.tif]

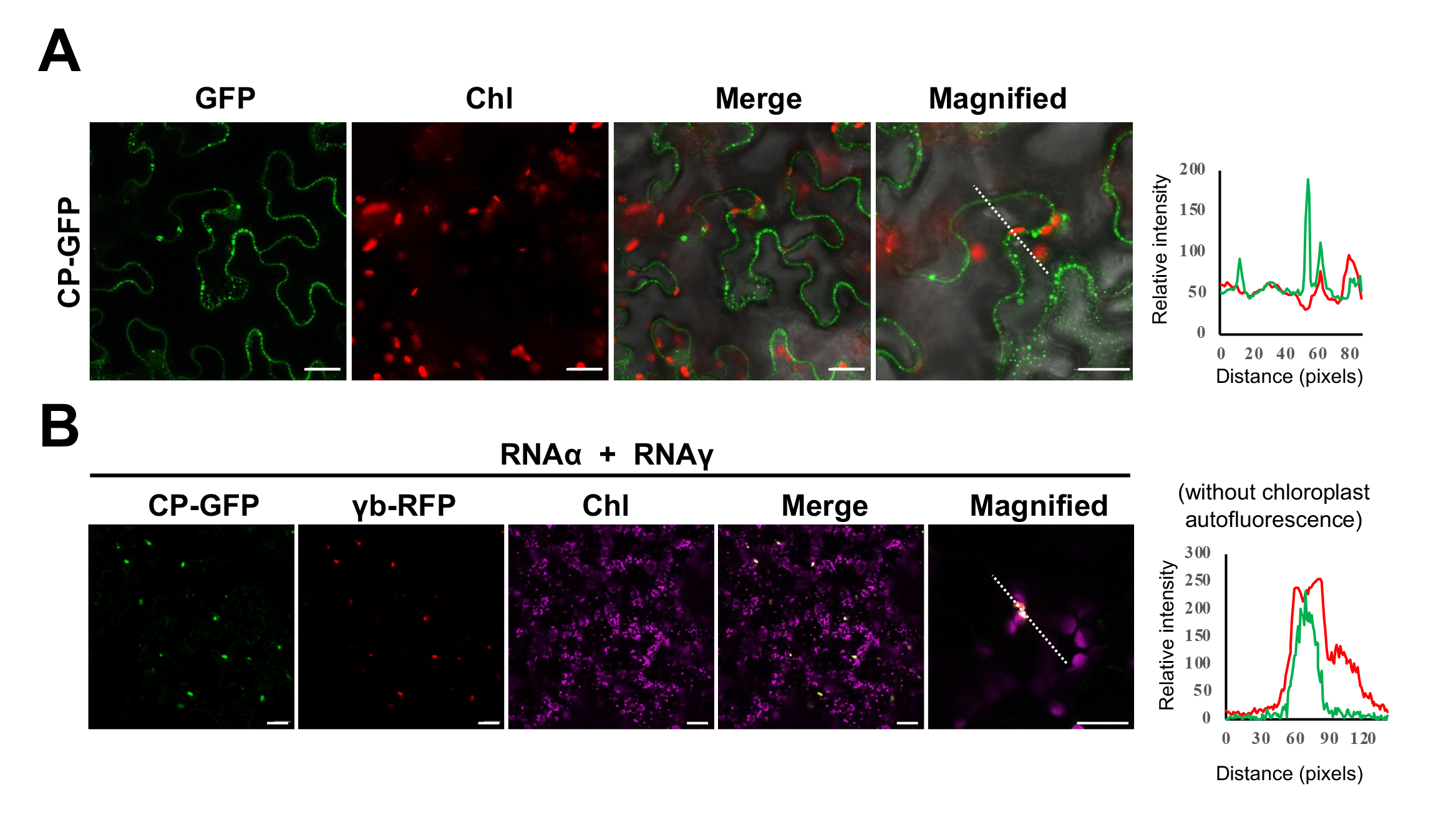

Supplement: S8 Fig — (A) Chloroplast autofluorescence is depicted as a false red color. Figure on the right indicate the normalized fluorescence intensities of the GFP (green) or the chloroplast autofluorescence (red) channels along the dashed white lines shown in the merged images of the magnified panel. Scale bars, 20 μm. (B) Co-localization analyses of CP-GFP and γb-RFP in the movement-deficient BSMV mutant (RNAα + RNAγ)-infiltrated leaves at 3 dpi. Chloroplast autofluorescence is depicted as a false pink color. Figures on the right indicate the normalized fluorescence intensity of GFP and RFP channels along the white dashed line in the merged confocal images. Scale bars, 20 μm. (TIF) [file ppat.1012311.s008.tif]

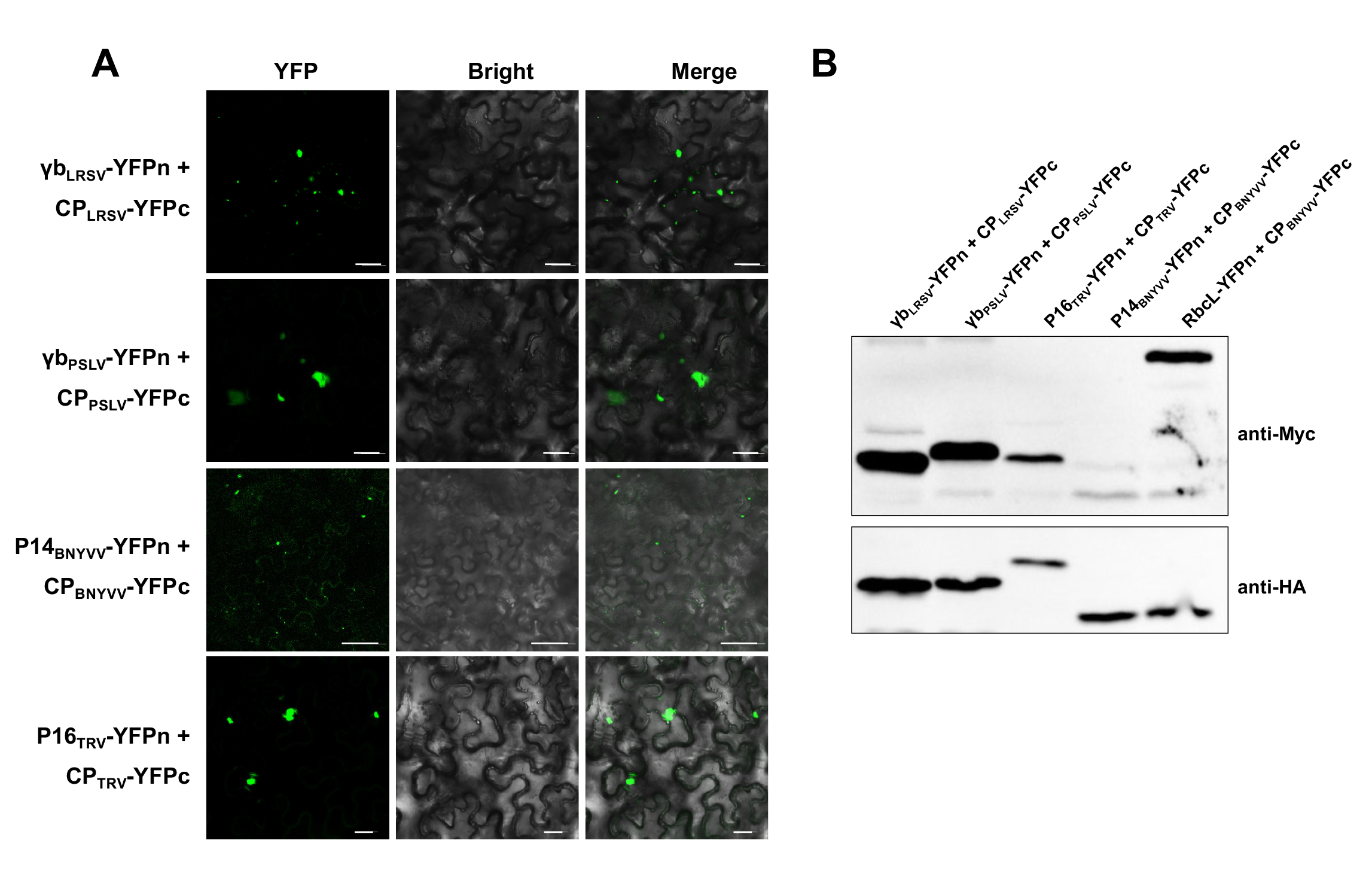

Supplement: S9 Fig — (A) BiFC assays to detect interactions between CRPs and CP of diverse viruses (LRSV, PSLV, TRV, and BNYVV). The combinations were shown on the left. N. benthamiana epidermal cells were observed by confocal microscope at 3 dpi. Scale bars, 20 μm. (B) Western blot to detect the proteins expression in S7A Fig. YFPn-fused proteins were identified with anti-Myc antibodies and YFPc-fused proteins were identified with anti-HA antibodies. (TIF) [file ppat.1012311.s009.tif]

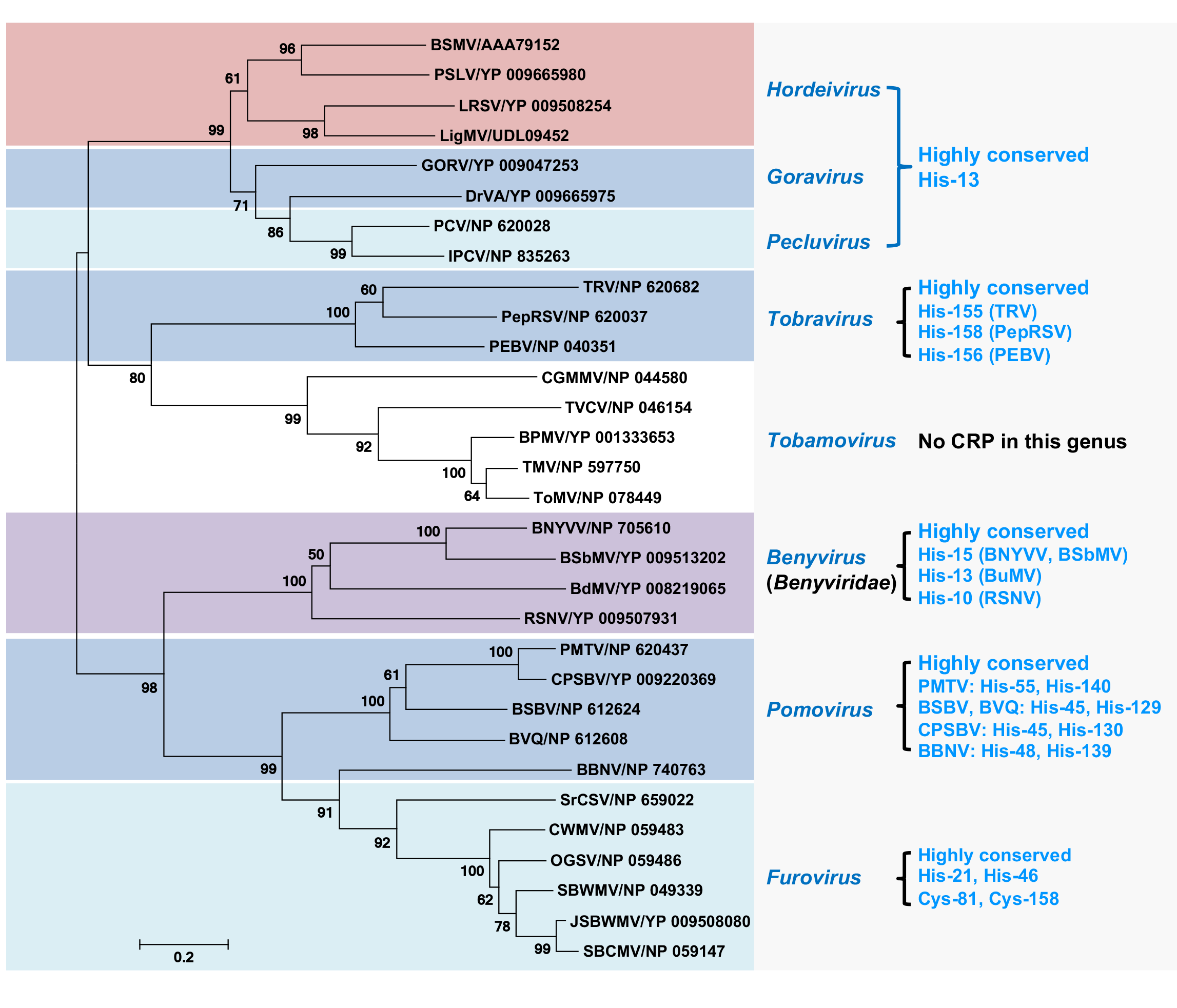

Supplement: S10 Fig — GenBank accession numbers of different CP proteins used for the construction of the phylogenetic tree are shown in the figure and available in the National Center for Biotechnology Information (NCBI) (https://www.ncbi.nlm.nih.gov/). The conserved Histidine site is marked on the right according to sequence analysis. The phylogenetic analyses were performed with the text neighbor-joining algorithm implemented in the MEGA5 software. (TIF) [file ppat.1012311.s010.tif]
