# Supplementary material for: Zn2+-dependent association of cysteine-rich protein with virion orchestrates morphogenesis of rod-shaped viruses
Source: PLoS Pathog. 2024 Jun 17;20(6):e1012311. doi: 10.1371/journal.ppat.1012311 (PMC11213338; doi:10.1371/journal.ppat.1012311)
Supplement: S1 Table — (DOCX) [file ppat.1012311.s011.docx]

**S1 Table.** Viral proteins identified by LC-MS/MS in purified BSMV virions after SDS-PAGE.

| **Bands** | **GenBank**  **Accession** | **Score ^a^** | **Mass**  **(Dalton)** | **Number of matches** | **Number of significant matches** | **Number of sequences** | **Number of significant sequences** | **emPAI** | **Sequence coverage (%)** | **Description** |
| --- | --- | --- | --- | --- | --- | --- | --- | --- | --- | --- |
| **A** | AAA79152 | 591 | 22538 | 37 | 18 | 15 | 8 | 3.4 | 44 | BSMV CP |
|  | AAA79153 | 143 | 56975 | 14 | 6 | 6 | 3 | 0.25 | 8 | BSMV TGB1 |
|  | AAA66599 | 53 | 17864 | 6 | 2 | 4 | 2 | 0.59 | 14 | BSMV γb |
| **B** | AAA79153 | 1993 | 56975 | 66 | 53 | 19 | 16 | 2.27 | 28 | BSMV TGB1 |
|  | AAA79152 | 800 | 22538 | 44 | 27 | 16 | 9 | 4.3 | 46 | BSMV CP |
|  | AAA66599 | 145 | 17864 | 9 | 4 | 4 | 2 | 0.59 | 18 | BSMV γb |
| **C** | AAA79152 | 1642 | 22538 | 86 | 50 | 18 | 13 | 10.1 | 59 | BSMV CP |
|  | AAA79153 | 170 | 56975 | 20 | 11 | 9 | 7 | 0.68 | 15 | BSMV TGB1 |
|  | AAA66599 | 94 | 17864 | 5 | 3 | 2 | 1 | 0.26 | 7 | BSMV γb |
| **D** | AAA79152 | 5777 | 22538 | 272 | 184 | 21 | 18 | 27.1 | 76 | BSMV CP |
|  | AAA66599 | 152 | 17864 | 8 | 4 | 4 | 2 | 0.59 | 13 | BSMV γb |

^a^ Individual ions scores > 43 indicate identity or extensive homology (*p* < 0.05).
