# Supplementary material for: Zn2+-dependent association of cysteine-rich protein with virion orchestrates morphogenesis of rod-shaped viruses
Source: PLoS Pathog. 2024 Jun 17;20(6):e1012311. doi: 10.1371/journal.ppat.1012311 (PMC11213338; doi:10.1371/journal.ppat.1012311)
Supplement: S2 Table — (DOCX) [file ppat.1012311.s012.docx]

**S2 Table.** Primers used for plasmid constructions and RT-qPCR analysis in this work.

| **Primer** | **Primer sequence (5'-3')** | **Purpose** | **Experiments** |
| --- | --- | --- | --- |
| γb-H85A-F | CAAAAGGCTGCGGATCTGTATGATTC | pCB301-γ_H85A,_ pGEXKG-γb_H85A_ pSPYNE-35S-γb_H85A,_ pSPYCE-35S-γb_H85A_ | Western blot |
| γb-H85A-R | GATCCGCAGCCTTTTGGCCACAGAACC |  |  |
| pET30a-LRSVγb-F | GGAATTCCATATGTGGCATCTTTAATG | pET30a-LRSVγb |  |
| pET30a-LRSVγb-R | CTCGAGCGGTTAGAACTATTGCGGAG |  |  |
| pET30a-PSLVγb-F | GGAATTCCATATGATGTCAACCGACTTGTGTTC | pET30a-PSLVγb |  |
| pET30a-PSLVγb-R | CTCGAGCGGGAGTTTACTTAGTTTGAAAAAATC |  |  |
| CPH13A-F | GCTAAGGGTGGAGGAGCCTACAACGAGGATCAATGGGA | pCB301-β_H13A,_ pET30a-CP_H13A_ |  |
| CPH13A-R | TTGATCCTCGTTGTAGGCTCCTCCACCCTTAGCAGTCA |  |  |
| pETGFP-γb-F | CTTTAAGAAGGAGATATACATATGATGATGGCTACTTTCTCTTG | pET30a-γb-GFP-His | MST assay |
| pETGFP-γb-R | CTCGCCCTTGCTCACCATCATATGTCCTCCGCCCTTAGAAACGGAAGAAGAATC |  |  |
| pETGFP-PSLVγb-R | CTCGCCCTTGCTCACCATCATATGTCCTCCGCCGAGTTTACTTAGTTTGAAAAAATCCG |  |  |
| BiFC-TRVp16-F | CCTGGCGCGCCACTAGTGGATCCATGACGTGTGCACTCAAGGG | pSPYNE-35S-P16_TRV_ | BiFC assay |
| BiFC-TRVp16-R | GTCGACAGTACTATCGATGGATCCAAAAGCAAACAACTGATCAATTC |  |  |
| BiFC-TRVCP-F | CCTGGCGCGCCACTAGTGGATCCATGGGAGATATGTACGATGAATC | pSPYNE-35S-CP_TRV_ |  |
| BiFC-TRVCP-R | GTCGACAGTACTATCGATGGATCCGGGATTAGGACGTATCGGACCTCC |  |  |
| BiFC-PSLV-γb-F | CCTGGCGCGCCACTAGTGGATCCATGTCAACCGACTTGTGTTC | pSPYNE-35S-γb_PSLV_ |  |
| BiFC-PSLV-γb-R | GTCGACAGTACTATCGATGGATCCGAGTTTACTTAGTTTGAAAAAATC |  |  |
| BiFC-LRSV-γb-F | CCTGGCGCGCCACTAGTGGATCCATGGCATCTTCACCTAATG | pSPYNE-35S-γb_LRSV_ |  |
| BiFC-LRSV-γb-R | GTCGACAGTACTATCGATGGATCCAAGCTTAGAACTATTGCGGAGAGC |  |  |
| BiFC-PSLV-CP-F | CCTGGCGCGCCACTAGTGGATCCATGCCGAACATCTCTCTTAC | pSPYNE-35S-CP_PSLV_ |  |
| BiFC-PSLV-CP-R | GTCGACAGTACTATCGATGGATCCAACCCGCTGACCTTGAGCCTG |  |  |
| BiFC-LRSV-CP-F | CCTGGCGCGCCACTAGTGGATCCATGGCTAATCTTGGACTCAC | pSPYNE-35S-CP_LRSV_ |  |
| BiFC-LRSV-CP-R | GTCGACAGTACTATCGATGGATCCAGCATTGTCTCCCGGAGCTC |  |  |
| BiFC-BNYVV-p14-F | CCTGGCGCGCCACTAGTGGATCCATGGGGATGGTAGATAGTTTG | pSPYNE-35S-p14_BNYVV_ |  |
| BiFC-BNYVV-p14-R | GTCGACAGTACTATCGATGGATCCCACTTCAGGATCGACAATAAC |  |  |
| BiFC-BNYVV-CP-F | CCTGGCGCGCCACTAGTGGATCCATGTCGAGTGAAGGTAGA | pSPYNE-35S-CP_BNYVV_ |  |
| BiFC- BNYVV-CP-R | GTCGACAGTACTATCGATGGATCCCTATTGTCCGGGTGGACTGG |  |  |
